# Detection of Antithrombotic-Related Bleeding in Older Inpatients: Multicenter Retrospective Study Using Structured and Unstructured Electronic Health Record Data

**DOI:** 10.2196/77809

**Published:** 2026-01-29

**Authors:** Claire Coumau, Frederic Gaspar, Mehdi Zayene, Elliott Bertrand, Lorenzo Alberio, Christian Lovis, Patrick E Beeler, Fabio Rinaldi, Monika Lutters, Marie-Annick Le Pogam, Chantal Csajka, SwissMADE Collaborators, Bernard Burnand

**Affiliations:** 1 Center for Research and Innovation in Clinical Pharmaceutical Sciences, Lausanne University Hospital and University of Lausanne, Rue du Bugnon 19, Lausanne, Switzerland, 1 021 314 42 63; 2 School of Pharmaceutical Sciences, University of Geneva, Geneva, Switzerland; 3 Institute of Pharmaceutical Sciences of Western Switzerland, University of Geneva, Geneva, Switzerland; 4 Artefact Company, Lausanne, Switzerland; 5 Service of Haematology and Central Haematology Laboratory, Lausanne University Hospital and University of Lausanne, Lausanne, Switzerland; 6 Division of Medical Information Sciences, Geneva University Hospitals and University of Geneva, Geneva, Switzerland; 7 Division of Occupational and Environmental Medicine, Epidemiology, Biostatistics and Prevention Institute, University of Zurich and University Hospital Zurich, Zurich, Switzerland; 8 Center for Clinical Research, University of Lucerne, Lucerne, Switzerland; 9 Dalle Molle Institute for Artificial Intelligence Research, IDSIA USI-SUPSI, Lugano, Switzerland; 10 SIB Swiss Institute of Bioinformatics, Lausanne, Switzerland; 11 Faculty of Biology and Medicine, Fondazione Bruno Kessler, Trento, Italy; 12 Head of Hospital Pharmacy, Kantonsspital Aarau, Aarau, Switzerland; 13 Department of Epidemiology and Health Systems, Unisanté, University Center for Primary Care and Public Health & University of Lausanne, Lausanne, Switzerland; 14 See Acknowledgments

**Keywords:** adverse drug events, adverse drug reactions, older inpatients, structured data mining, machine learning, natural language processing, electronic medical records, multicenter study, antithrombotic, hemorrhage, artificial intelligence, pharmacovigilance

## Abstract

**Background:**

Bleeding complications are a major contributor to adverse drug events among older inpatients, particularly in those treated with antithrombotic agents. Timely and accurate detection of bleeding events is essential for improving drug safety surveillance and clinical risk management.

**Objective:**

The study aimed to develop and validate automated algorithms for detecting major bleeding (MB) and clinically relevant nonmajor bleeding (CRNMB) events from electronic medical records (EMRs) by combining structured data-based rule models and a natural language processing (NLP) approach, and to evaluate their performance and generalizability against a manually reviewed gold standard and an external dataset.

**Methods:**

We conducted a multicenter retrospective study using routinely collected EMR data from 3 Swiss university hospitals. Patients 65 years or older who received at least one antithrombotic agent and were hospitalized between January 2015 and December 2016 were included. To detect MB and CRNMB events, rule-based algorithms were developed using structured data (*International Statistical Classification of Diseases, 10th Revision, German Modification* [*ICD-10-GM*] codes, laboratory values, transfusion records, and antihemorrhagic prescriptions), with variables and cutoff values defined according to adapted International Society on Thrombosis and Haemostasis definitions and expert consensus. In parallel, a supervised NLP model was applied to discharge summaries from one hospital. A manual review of 754 EMRs served as the reference standard for internal validation, and the algorithm performance of the structured data algorithms (SDA), NLP, and their combination (SDA+NLP) was evaluated against this manually reviewed gold standard using standard performance metrics. External validation was performed on an independent dataset from the Lausanne University Hospital to assess model robustness and generalizability.

**Results:**

Among 36,039 inpatient stays, SDA identified 8.26% (n=2979) as MB and 15.04% (n=5419) as CRNMB cases. *ICD-10-GM* codes alone detected 28.5% (n=849) of MB and 31.48% (n=1706) of CRNMB cases, while laboratory data contributed most to event detection (n=1994, 66.94% for MB and n=3663, 67.60% for CRNMB). Integrating SDA with NLP improved detection, identifying 12.2% (920/7513) of MB and 27.4% (2062/7513) of CRNMB cases at 1 hospital. The combined model achieved the best performance (sensitivity 0.84, positive predictive value 0.51, *F*_1_-score 0.64). External validation on Lausanne University Hospital 2021‐2022 data (n=24,054 stays) confirmed the algorithms’ reproducibility; the prevalence of MB decreased while CRNMB increased, reflecting evolving clinical practices and antithrombotic use patterns.

**Conclusions:**

Our integrated approach, combining SDA with NLP, enhances the detection of hemorrhagic events in older hospitalized patients treated with antithrombotic agents, suggesting its potential usefulness for drug safety monitoring and clinical risk management.

## Introduction

Over 16% of older inpatients experience at least 1 adverse drug event (ADE) during their hospital stay [1], often with more severe consequences than in younger patients [2]. Among the medications most frequently implicated, antithrombotic agents, widely prescribed in older adults for the prevention and treatment of cardiovascular disease, stand out as a major cause of bleeding-related ADEs [1,3]. Hemorrhagic complications represent a substantial share of drug-related harm in this population and are associated with longer hospital stays, higher readmission rates, and increased mortality. Continuous and accurate measurement of these events is therefore essential to inform prevention strategies, strengthen pharmacovigilance, and promote safer antithrombotic use in clinical practice.

Various approaches have been developed to detect ADEs in hospital settings, each with advantages and limitations. Spontaneous reporting systems, though simple to implement, notoriously underestimate the true frequency of ADEs due to underreporting [4]. Systematic chart reviews of electronic medical records (EMRs), often considered the reference standard, provide detailed clinical information but are too resource- and time-intensive for routine surveillance [5]. To overcome these constraints, automated detection methods using routinely collected EMR data have emerged. These approaches leverage both structured data, such as diagnostic codes, medication records, laboratory results, and vital signs, and unstructured clinical narratives, including discharge summaries, progress notes, and consultation reports. Structured data are accessible and standardized, supporting large-scale analyses but may lack contextual nuances needed to capture complex clinical events such as bleeding [6-8]. Conversely, textual data, although unstructured, often contain richer clinical detail but require advanced computational methods for analysis. Recent advances in machine learning (ML) and natural language processing (NLP) have markedly improved the ability to extract this information and are increasingly applied to pharmacovigilance and ADE detection [9]. Integrating both structured and textual data appears particularly promising for identifying bleeding events, potentially enhancing accuracy and completeness [10].

Despite growing interest in automated ADE detection to support drug safety monitoring, important knowledge gaps remain, particularly in the Swiss context. Most existing studies focusing on bleeding events have relied exclusively on either structured or unstructured data [11-15], have prioritized prediction rather than detection [8,16], or have focused on specific bleeding types or patient groups [7,10,17-19]. Furthermore, clear operational definitions distinguishing major bleeding (MB) from clinically relevant nonmajor bleeding (CRNMB) are often lacking, limiting comparability across studies [20]. To date, no study in Switzerland has comprehensively evaluated the combined contribution of structured and textual data for ADE detection in a general inpatient population receiving antithrombotic therapy. To address this gap, we conducted a multicenter study integrating rule-based algorithms and NLP to detect MB and CRNMB events among older inpatients treated with antithrombotics. We hypothesized that combining structured and textual EMR data would improve the accuracy and completeness of bleeding event identification compared with using either data source alone. The study aimed to develop rule-based algorithms for bleeding detection from structured data sources (diagnoses, laboratory results, transfusions, and antihemorrhagic prescriptions) based on international definitions; design and train an NLP model to identify bleeding mentions in discharge summaries; assess and compare the diagnostic performance of structured data algorithms (SDA), NLP, and their combination (SDA+NLP) against a manually reviewed gold standard; and evaluate the generalizability of the best-performing models through external validation on an independent dataset.

## Methods

### Study Design

We conducted a multicenter cross-sectional study using retrospective data covering the period from January 1, 2015, to December 31, 2016. Data were obtained from 4 large Swiss hospitals: Lausanne University Hospital (CHUV; approximately 1500 beds [21]), Geneva University Hospital (HUG; approximately 2000 beds [22]), both located in the French-speaking region and serving the cantons of Vaud and Geneva, respectively, Zürich University Hospital (USZ; approximately 900 beds [23]) serving the Zurich metropolitan area, and Baden Cantonal Hospital (KSB; approximately 400 beds [24]) serving the canton of Aargau in the German-speaking region. This study was conducted in accordance with the SRTOBE (Strengthening the Reporting of Observational Studies in Epidemiology) statement (Checklist 1).

The 2015‐2016 dataset was used for algorithm development as it was the most recent period with harmonized, high-quality structured and unstructured EMR data across all hospitals. Later years were excluded due to EMR vendor transitions, database restructuring, and new data-governance restrictions limiting access to deidentified text. A more recent CHUV dataset (2021‐2022) was used for temporal and external validation to test algorithm robustness under evolving clinical practices and documentation standards.

### Study Participants and Hospital Stays

Eligible participants were Swiss residents 65 years or older treated with at least 1 antithrombotic agent during their hospital stay. Antithrombotic agents included vitamin K antagonists, heparins, platelet aggregation inhibitors, direct thrombin inhibitors, direct factor Xa inhibitors, or fondaparinux. Hospitalizations had to last at least 24 hours and to occur between January 2015 and December 2016 (test dataset). For the external validation, an additional dataset from CHUV covering January 2021 to December 2022 was used (validation dataset). Only patients who had provided explicit consent for the reuse of their health data for research purposes, as indicated by the signature of the general consent form, were eligible for inclusion. Hospital stays lasting less than 24 hours were excluded from the analysis.

### Data Sources and Preprocessing

Each participating hospital extracted relevant clinical data from its institutional data warehouses for all inpatient stays meeting the inclusion criteria. The extracted datasets included both structured and unstructured data. Structured data comprised administrative information, patient movements within the hospital, key clinical and laboratory parameters, and prescribed medications coded using the anatomical therapeutic chemical classification. Diagnostic codes were drawn from the *International Statistical Classification of Diseases, 10th Revision, German Modification* (*ICD-10-GM*), and procedures were coded according to the Swiss Classification of Surgical Procedures (CHOP). Diagnoses and procedures were obtained from the hospital billing records associated with each inpatient stay. Unstructured data included discharge summaries. Further details on data extraction and handling are available in the published study protocol [25].

**Figure 1.**
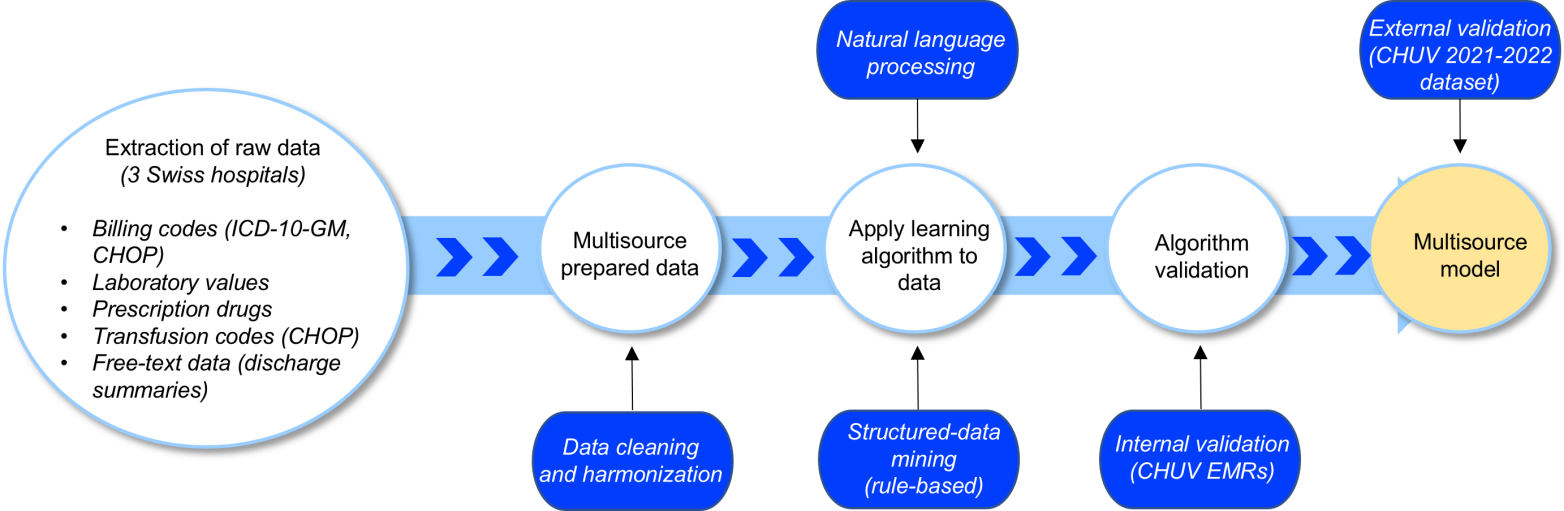
Overview of the data extraction and preprocessing pipeline for structured and unstructured electronic medical record (EMR) data. CHOP: Swiss Classification of Surgical Procedures; CHUV: Lausanne University Hospital; *ICD-10-GM*: *International Statistical Classification of Diseases, 10th Revision, German Modification*.

Prior to analysis, structured data were cleaned, harmonized, and verified for consistency at each site, then locally deidentified before being transferred to a centralized database hosted at CHUV. Unstructured data were deidentified and, where necessary, converted into machine-readable formats, but were stored locally on secure hospital servers to comply with data governance policies. Due to the extent of missing and inconsistent information, such as discrepancies in data structure, coding systems, variable definitions, and extensive missing values, reliable harmonization of KSB data with the other hospitals was not feasible, and data from KSB were excluded from the analysis. In addition, only unstructured data from CHUV were analyzed, as full deidentification of textual data from the other sites could not be ensured. The same preprocessing workflow was applied to the 2021‐2022 CHUV dataset used for external validation. An overview of the data processing workflow is provided in Figure 1.

### Bleeding Detection Algorithms

**Figure 2.**
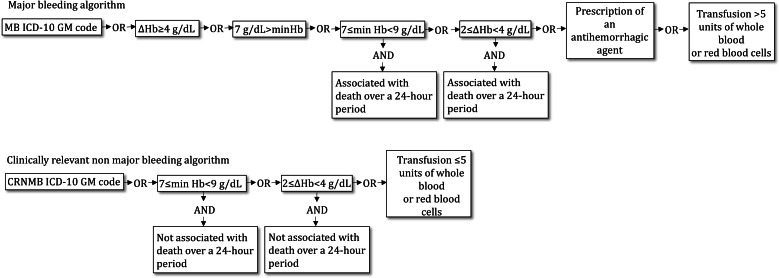
Algorithmic framework for detection of major bleeding (MB) and nonmajor clinically relevant bleeding (CRNMB) cases using structured data. Antihemorrhagic agent: idarucizumab, andexanet alfa, prothromplex, octaplex, and beriplex. ∆Hb: drop in hemoglobin levels within 48 hours; *ICD-10-GM*: *International Statistical Classification of Diseases, 10th Revision, German Modification*; Min Hb: minimum hemoglobin value during the stay.

We selected variables of interest and cutoff values for our algorithms based on an adaptation of the International Society on Thrombosis and Haemostasis (ISTH) definitions of MB [26] and CRNMB [27], informed by an extensive review of international guidelines (Multimedia Appendix 1). MB was defined as a hemoglobin drop of 4 g/dL or more within 48 hours, a 2 to 4 g/dL drop associated with death within 24 hours, a hemoglobin level less than 7 g/dL, a hemoglobin level between 7 and 9 g/dL associated with death within 24 hours, or a transfusion of more than 5 units of blood or red blood cells. CRNMB was defined as a hemoglobin drop of 2 to 4 g/dL within 48 hours not associated with death or a hemoglobin nadir between 7 and 9 g/dL without subsequent death. The ISTH hemoglobin thresholds were adapted to improve specificity in older inpatients and to reduce the risk of misclassifying nonhemorrhagic anemias. The 5-unit transfusion threshold was pragmatically chosen due to the limited granularity of CHOP procedural codes. Additional structured indicators were also integrated to refine case classification: the prescription of antihemorrhagic agents (idarucizumab, andexanet alfa, prothromplex, octaplex, and beriplex) was considered indicative of MB. MB-related in-hospital mortality was defined as any hospital stay involving at least 1 MB event followed by death during the same admission. We then developed rule-based algorithms using Boolean logic to detect MB and CRNMB cases from structured data (Figure 2).

The *ICD-10-GM* list comprised 12 codes for MB and 41 codes for CRNMB (Table 1). We defined an MB in-hospital mortality case as a stay containing an MB occurring during hospitalization followed by death of the patient during the same hospitalization period. We measure the prevalence of bleeding cases, corresponding to inpatient stays with at least 1 MB or CRNMB event, either present on admission or occurring during hospitalization. We quantified the relative and absolute contribution of each structured data source (diagnoses, laboratory, transfusions, and medications), both individually and in combination, in terms of overall detection capacity and proportion of identified bleeding events.

**Table 1.** Lists of deficient systems/organs and distribution of *ICD-10-GM*^a^ chapters and codes identifying MB^b^ and CRNMB^c^ cases.

Types of hemorrhagic events	*ICD-10-GM* codes
MB	
Hyphema	H21.0
Hemorrhage and rupture of the choroid	H31.3
Retinal, vitreous, or subarachnoid hemorrhage	H35.6, H43.1, I60_
Hemopericardium not classified elsewhere	I31.2
Intracerebral hemorrhage	I61_
Other nontraumatic intracranial hemorrhages	I62_
Hemoperitoneum	K66.1
Hemarthrosis	M25.0_
Hypovolemic shock	R57.1
Shock during or after a procedure for diagnostic and therapeutic purposes, not classified elsewhere	T81.1
CRNMB	
Conjunctival hemorrhage	H11.3
Otorrhagia	H92.2
Hemorrhagic esophageal varices	I85.0, I98.3
Other specified diseases of the esophagus	K22.8
Non-traumatic hemothorax	J94.2
Gastric, duodenal, or gastrojejunal ulcer with hemorrhage and/or perforation	K25.0, K25.2, K25.6, K26.0, K26.2, K26.4, K26.6, K27.0, K27.2, K27.4, K27.6, K28.0, K28.2, K28.4, K28.6
Acute hemorrhagic gastritis	K29.0
Rectal and anal hemorrhage	K62.5
Hematemesis	K92.0
Melena	K92.1
Unspecified gastrointestinal hemorrhage	K92.2
Prostatic congestion and hemorrhage	N42.1
Hematoma of the broad ligament	N83.7
Hematometra	N85.7
Abnormal bleeding from the uterus and vagina	N93.8, N93.9
Postmenopausal bleeding	N95.0
Epistaxis	R04.0
Throat hemorrhage	R04.1
Hemoptysis	R04.2
Respiratory tract hemorrhage	R04.8, R04.9
Spontaneous ecchymosis	R23.3
Unspecified hematuria	R31
Hemorrhage, not classified elsewhere	R58, T81.0

a *ICD-10-GM*: *International Classification of Diseases, 10th Revision, German Modification*.

b MB: major bleeding.

c CRNMB: clinically relevant nonmajor bleeding.

### Natural Language Processing Model

To complement structured data detection, we developed a supervised ML model to identify MB, CRNMB, and past bleeding cases documented in discharge summaries.

**Figure 3.**
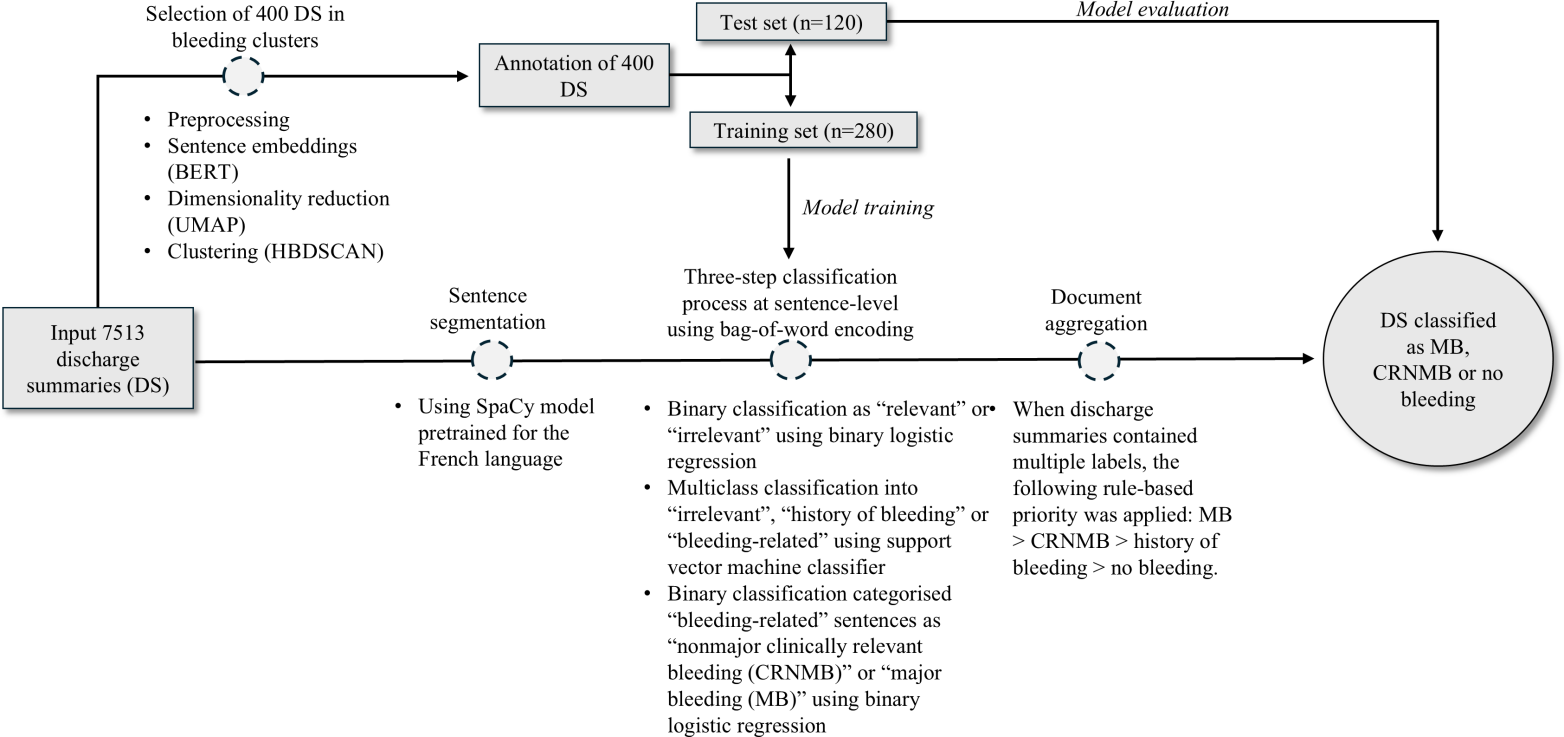
Natural language processing workflow from raw text input to final classification output. BERT: Bidirectional Encoder Representations From Transformers; DS: discharge summary; HBDSCAN: Hierarchical Density-Based Spatial Clustering of Applications With Noise; UMAP: Uniform Manifold Approximation and Projection.

A dataset of 400 discharge summaries from CHUV was randomly divided into a training set (n=280) and a test set (n=120), including 100 summaries with MB, 100 with CRNMB, and 200 with no bleeding. Three independent physicians manually annotated the 400 discharge summaries using 4 mutually exclusive labels: (A) ‘presence of CRNMB,’ (B) ‘presence of MB’ (as previously defined), (C) ‘history of bleeding’ (when a discharge summary mentioned bleeding in the EMR before the hospital admission), and (D) ‘absence of any bleeding.’ Preprocessing steps included tokenization, lemmatization, and sentence segmentation using the French spaCy model (v3.0) [28]. The classification pipeline combined logistic regression and support vector machine models, selected for their interpretability and robustness with limited training data. We deliberately used a classical supervised ML model rather than deep learning architectures to ensure interpretability, reproducibility, and computational efficiency, which are essential for clinical validation and routine pharmacovigilance applications. This approach also better suited the relatively small, annotated corpus, allowing transparent feature weighting and easier auditability across institutions. The model was trained using the scikit-learn library (Python v3.9.1). The classification pipeline proceeded in 3 stages: step 1: binary classifier to identify bleeding-relevant versus irrelevant sentences; step 2: multiclass classifier to distinguish between *irrelevant*, *antecedent bleeding*, and *active bleeding*; step 3: binary classifier to further differentiate between MB and CRNMB within sentences flagged as active bleeding. Sentence-level predictions were aggregated to assign a final label to each document. Rules were prioritized as follows: MB>CRNMB>history of bleeding>no bleeding. This ensured a conservative classification hierarchy, favoring the identification of more severe bleeding cases when multiple labels were present. Further methodological details are available in the study proposal previously published [25], the related article [29], and summarized in Figure 3.

### Validation of the Bleeding Detection Algorithms

#### Internal Validation Using CHUV 2015-2016 Data

To validate the SDA and SDA combined with NLP (SDA+NLP) models, we conducted a manual review of 754 EMRs from CHUV’s 2015‐2016 dataset. The sample size for validation was determined using a test result-based sampling method [30]. Assuming a 7% MB, a 10% CRNMB accuracy, and a sensitivity of 0.7, at least 704 EMRs had to be reviewed, and 754 EMRs were effectively reviewed. Four physicians independently reviewed the records to compare algorithm-detected with clinician-identified MB and CRNMB cases. The review process followed a structured protocol aligned with ISTH definitions [26,27] and adapted for retrospective application to routinely collected hospital data. Reviewers assessed each inpatient stay according to 4 key criteria: (1) evidence of active bleeding, (2) severity of the event (eg, hemodynamic instability), (3) need for therapeutic intervention (eg, transfusion volume, administration of antihemorrhagic agents), and (4) temporal relationship to hospital admission (present on admission versus occurred during stay). A complete list of synonyms used to identify MB and CRNMB cases during manual chart review is provided in Multimedia Appendix 2. Two binary classification scenarios were evaluated: (1) MB versus all other cases (CRNMB or no bleeding), and (2) CRNMB versus no bleeding (excluding MB). Algorithm performance was evaluated at the inpatient-stay level using standard binary classification metrics (sensitivity, specificity, positive predictive value [PPV], negative predictive value, accuracy, and *F*_1_-score), with manual chart review as the gold standard. Comparisons between SDA, NLP, and combined models were descriptive, and sensitivity was prioritized due to the study’s patient safety focus. Interrater reliability among reviewers was evaluated using Fleiss κ on a subset of 40 cases, with agreement levels interpreted according to Landis and Koch [31] (>0.80: *almost perfect*; 0.61‐0.80: *substantial*; 0.41‐0.60: *moderate*; 0.21‐0.40: *fair*; 0.00‐0.20: *slight*; <0.00: *poor*). A *P* value associated with the Fleiss κ coefficient was also calculated, with a *P* value less than .05 indicating statistically significant agreement. Additional details and results are provided in Multimedia Appendix 3. In a subanalysis, a causal relationship between antithrombotic therapy and each bleeding event was also assessed during the manual review, using a structured tool based on temporal association, biological plausibility, and alternative explanations. Cases were rated as *certain*, *probable*, *possible*, or *unclassified,* in relation to antithrombotic exposure, according to the WHO-Uppsala Monitoring Center scale [32]. The methodology, sample size calculation, and findings of the causality assessment of the subanalysis are presented in Multimedia Appendix 4.

#### External Validation Using CHUV 2021-2022 Data

An external validation was performed using CHUV data from 24,054 inpatient stays between January 2021 and December 2022. We applied the same detection algorithms (SDA and SDA+NLP) to this independent dataset to evaluate their performance, robustness, and reproducibility. Results were compared to those from the 2015‐2016 CHUV dataset.

### Statistical Analysis

Descriptive statistics were used to summarize population characteristics. Comorbidity was assessed using the Charlson and Elixhauser indexes [33,34], which are validated tools for risk adjustment and mortality prediction based on administrative health data. Comparisons of patient characteristics between hospitals were conducted using a 1-way analysis of variance on ranks (Kruskal-Wallis test) for continuous variables and Pearson χ^2^ test for categorical variables. Hyperparameters of the NLP classifier were optimized through 5-fold cross-validation on the training set, and final performance was estimated on an independent test set. All performance metrics were reported with 95% CIs calculated using the Wilson method. Analyses were conducted using StataCorp. 2021. Stata Statistical Software: Release 17. College Station, TX: StataCorp LLC software for structured data and Python (v3.9.1) for NLP development.

### Ethical Considerations

#### Human Subject Ethics Review Approvals or Exemptions

This study was conducted in accordance with the *Declaration of Helsinki* and Swiss federal regulations governing research on human data. Ethical approval was obtained from all relevant cantonal ethics committees, coordinated by the lead committee of the Canton of Vaud (CER-VD No. 2018‐00272). As the study involved secondary analysis of routinely collected, deidentified hospital data, it qualified for a simplified review under Swiss Human Research Act article 2, paragraph 2(c).

#### Informed Consent

The study relied exclusively on existing clinical data that were deidentified before analysis. According to Swiss regulations and institutional data governance policies, informed consent was waived for patients who had not explicitly objected to the use of their medical data for research purposes. All participating hospitals operate an institutional opt-out procedure, allowing patients to refuse the secondary use of their data for research.

#### Privacy and Confidentiality

All data were deidentified at source before analysis. Structured data were transferred through secure institutional channels to a restricted-access research environment hosted at CHUV. Unstructured textual data remained stored locally on hospital servers and were processed within each institution’s secure infrastructure to comply with data protection requirements. No directly identifiable information was accessible to the investigators.

#### Compensation Details

No compensation was provided to patients, as the study involved secondary analysis of preexisting, routinely collected data and did not include direct contact with participants.

#### Protection of Identifiable Information in Figures and Supplementary Materials

No image, document, or figure contains any identifiable patient information. Consequently, no individual consent for image publication was required.

#### Ethics Approval

Approved by the Cantonal Ethics Committee of Vaud, Switzerland (CER-VD No. 2018‐00272); informed consent was waived for patients who did not opt out of research data use.

## Results

### Study Population Characteristics

A total of 36,039 inpatient stays, involving 24,991 unique patients, were included in the analysis: 7677 stays (5754 patients) at CHUV, 18,015 stays (11,356 patients) at HUG, and 10,347 stays (7881 patients) at USZ. Patient characteristics are detailed in Table 2. The median age at admission was 78 (IQR 65‐99) years, with a balanced sex distribution (51.40% male). Comorbidity was generally low across the cohort, with a median Charlson index and Elixhauser index of 0.0; USZ patients had the lowest overall comorbidity burden.

**Table 2.** Baseline patient characteristics and treatments: overall and by university hospital.

Characteristics	All hospitals (n=36,039)^a^	CHUV^b^ (n=7677)	HUG^c^ (n=18,015)	USZ^d^ (n=10,347)
Admission age (years), median (IQR)	78 (65‐99)	79 (65‐99)	80 (65‐99)	75 (65‐92)
Sex, n (%)				
Male	18,525 (51.40)	3987 (51.93)	8638 (47.95)	5900 (57.02)
Female	17,514 (48.60)	3690 (48.07)	9377 (52.05)	4447 (42.98)
Length of stay (d), median (IQR)	9 (1-342)	9 (1-293)	12 (1‐342)	6 (1-145)
Transfer to intensive care, n (%)	1534 (4.26)	467 (6.1)	1067 (5.92)	—^e^
In-hospital mortality, n (%)	1416 (3.93)	345 (4.5)	850 (4.7)	221 (2.1)
Comorbidity, n (%)				
Chronic renal dysfunction	8418 (23.36)	2163 (28.18)	5662 (31.43)	593 (5.7)
Dialysis	622 (1.7)	176 (2.3)	241 (1.3)	205 (2.0)
Acute renal dysfunction	1151 (3.19)	266 (3.5)	623 (3.5)	262 (2.5)
Chronic liver dysfunction	1020 (2.83)	294 (3.8)	497 (2.8)	229 (2.2)
Acute liver dysfunction	498 (1.4)	145 (1.9)	244 (1.4)	109 (1.1)
Hypertension	18,316 (50.82)	3158 (41.14)	9271 (51.46)	5887 (56.90)
Alcohol abuse	1354 (3.76)	388 (5.1)	663 (3.7)	303 (2.9)
Stroke	3001 (8.33)	813 (10.6)	1625 (9.02)	563 (5.4)
Cancer	6776 (18.80)	1572 (20.48)	2905 (16.13)	2299 (22.22)
Platelet coagulation defect	2178 (6.04)	496 (6.5)	1029 (5.71)	653 (6.3)
Anemia	7624 (21.15)	1998 (26.03)	4380 (24.31)	1246 (12.04)
Risk fall	11,376 (31.57)	2932 (38.20)	6021 (33.42)	2423 (23.42)
Diabetes	6638 (18.42)	1314 (17.12)	3575 (19.84)	1749 (16.90)
Recent myocardial infection	1923 (5.34)	609 (7.9)	761 (4.2)	553 (5.3)
Low weight	4059 (11.26)	967 (12.6)	2533 (14.06)	559 (5.4)
Thrombolysis	695 (1.9)	180 (2.3)	512 (2.8)	3 (0.0)
Vascular malformation	955 (2.6)	153 (2.0)	334 (1.9)	468 (4.5)
Charlson comorbidity index, median (IQR)	0.0 (0.0‐9.0)	0.0 (0.0‐9.0)	0.0 (0.0‐7.0)	0.0 (0.0‐7.0)
Elixhauser comorbidity index, median (IQR)	0.0 (0.0‐6.0)	0.0 (0.0‐6.0)	0.0 (0.0‐6.0)	0.0 (0.0‐5.0)
Antithrombotic categories, n (%)				
Direct factor Xa inhibitors	3297 (9.15)	599 (7.8)	1478 (8.20)	1220 (11.80)
Vitamin K antagonists	7469 (20.72)	1324 (17.25)	4943 (27.44)	1202 (11.62)
Heparin group	24,784 (6877)	5045 (65.71)	11,918 (66.17)	7821 (75.59)
Direct thrombin inhibitors	255 (0.7)	87 (1.1)	134 (0.7)	34 (0.3)
Platelet aggregation inhibitors	14,220 (39.46)	4354 (56.71)	4700 (26.09)	5166 (49.93)
Thrombolytics	104 (0.3)	15 (0.2)	89 (0.5)	0.0 (0.0)
Other antithrombotic agents: fondaparinux	1365 (3.79)	212 (2.8)	1140 (6.33)	13 (0.1)
Antidotes, n (%)	137 (0.4)	15 (0.2)	122 (0.7)	0.0 (0.0)
Transfusion, n (%)	582 (1.6)	264 (3.4)	318 (1.8)	—
≤5 UI^f^ plasma or red blood cells	225 (0.6)	100 (1.3)	125 (0.7)	—
>5 UI plasma or red blood cells	357 (1.0)	164 (2.1)	193 (1.1)	—
Number of antithrombotic agents received during hospitalization, n (%)				
1	22,397 (62.15)	4257 (55.45)	12,381 (68.73)	5759 (55.66)
2	11,918 (33.07)	2904 (37.83)	4924 (27.33)	4090 (39.53)
3	1641 (4.55)	495 (6.4)	669 (3.7)	477 (4.6)
≥4	83 (0.2)	21 (0.3)	41 (0.2)	21 (0.2)

a n: total number of recorded measurements for the respective parameter.

b CHUV: Lausanne University Hospital.

c HUG: Geneva University Hospital.

d USZ: Zürich University Hospital.

e Not available (missing or nontransferred data).

f UI: unit of blood component.

Distinct prescribing patterns were observed across hospitals: HUG had the highest use of vitamin K antagonists (n=4943, 27.44%), CHUV had the highest prescription rate of antiplatelet agents (n=4354, 56.71%), and USZ reported the highest use of direct factor Xa inhibitors (n=1220, 11.79%) and heparins (n=7821, 75.59%). Hypertension (n=18,316, 50.82%), chronic renal dysfunction (n=8418, 23.36%), anemia (n=7624, 21.15%), and cancer (n=6776, 18.80%) were among the most prevalent comorbidities. Overall, in-hospital mortality was 3.93% (n=1416).

### Bleeding Detection Using SDA

SDA detected 8748 (24.27%) overall bleeding cases, of which 2979 (8.26%) were MB cases and 5419 (15.04%) were CRNMB cases (Table 3). Fatal MB occurred in 1.0% (n=350) of all stays. MB prevalence varied across hospitals, with the highest proportion observed at CHUV (n=769, 10.0%), followed by USZ (n=998, 9.6%) and HUG (n=1212, 6.73%). CRNMB prevalence was highest at USZ (n=1682, 16.26%). Missing values for each variable used to identify MB and CRNMB events are presented in Multimedia Appendix 5.

**Table 3.** Prevalence of bleeding cases detected by SDA^a^, overall and by university hospital^b^.

	All hospitals, n (%)	CHUV^c^, n (%)	HUG^d^, n (%)	USZ^e^, n (%)	*P* value^f^
Nonbleeding-related	27,641 (76.70)	5822 (75.84)	14,152 (78.56)	7667 (74.10)	<.001
CRNMB^g^	5419 (15.04)	1086 (14.15)	2651 (14.72)	1682 (16.26)	<.001
MB^h^	2979 (8.26)	769 (10.0)	1212 (6.73)	998 (9.6)	<.001
MB in-hospital mortality	350 (1.0)	119 (1.6)	137 (0.8)	94 (0.9)	<.001
Total	36,039	7677	18,015	10,347	—^i^

a SDA: structured data algorithms (ie, rule-based algorithm for structured data).

b Bleeding cases: number of stays for patients treated with at least 1 antithrombotic agent during which at least 1 bleeding episode occurred.

c CHUV: Lausanne University Hospital.

d HUG: Geneva University Hospital.

e USZ: Zürich University Hospital.

f Using Pearson *χ*2 test.

g CRNMB: clinically relevant nonmajor bleeding.

h MB: major bleeding.

i Not applicable.

### Relative and Absolute Contribution of Structured Data Sources

**Figure 4.**
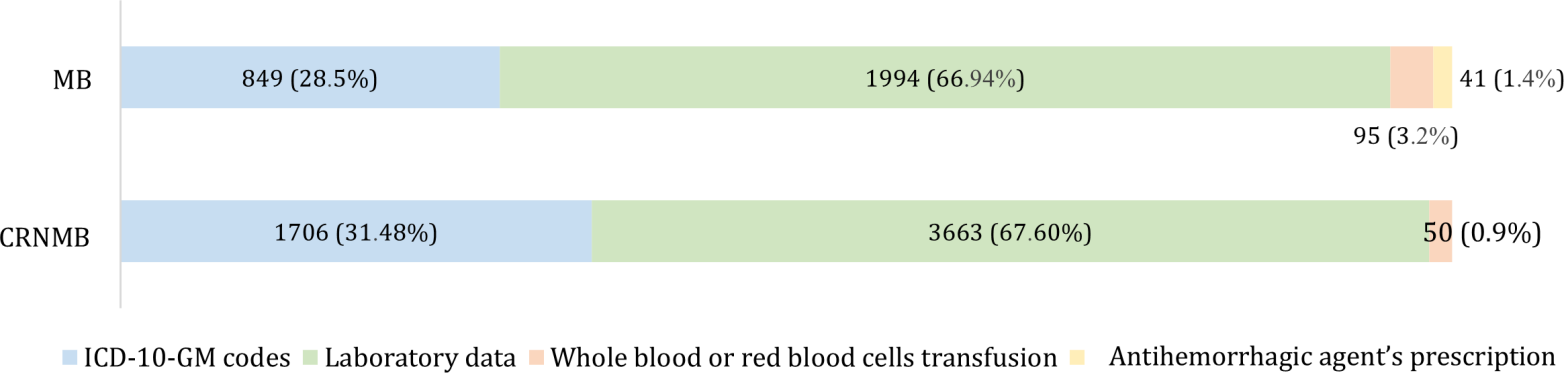
Relative contribution of structured data sources (laboratory data, *ICD-10-GM* codes, prescription of antihemorrhagic agents, and transfusions) to the detection of major bleeding (MB) and clinically relevant nonmajor bleeding (CRNMB). *ICD-10-GM*: *International Statistical Classification of Diseases, 10th Revision, German Modification*.

Laboratory data were the most influential source for detecting both MB and CRNMB, contributing to two-thirds of identified cases, while *ICD-10-GM* codes contributed to approximately one-third. Prescriptions for antihemorrhagic agents had a minimal added value for MB detection, while transfusion data contributed modestly. Figure 4 illustrates the relative contribution of each data source.

**Figure 5.**
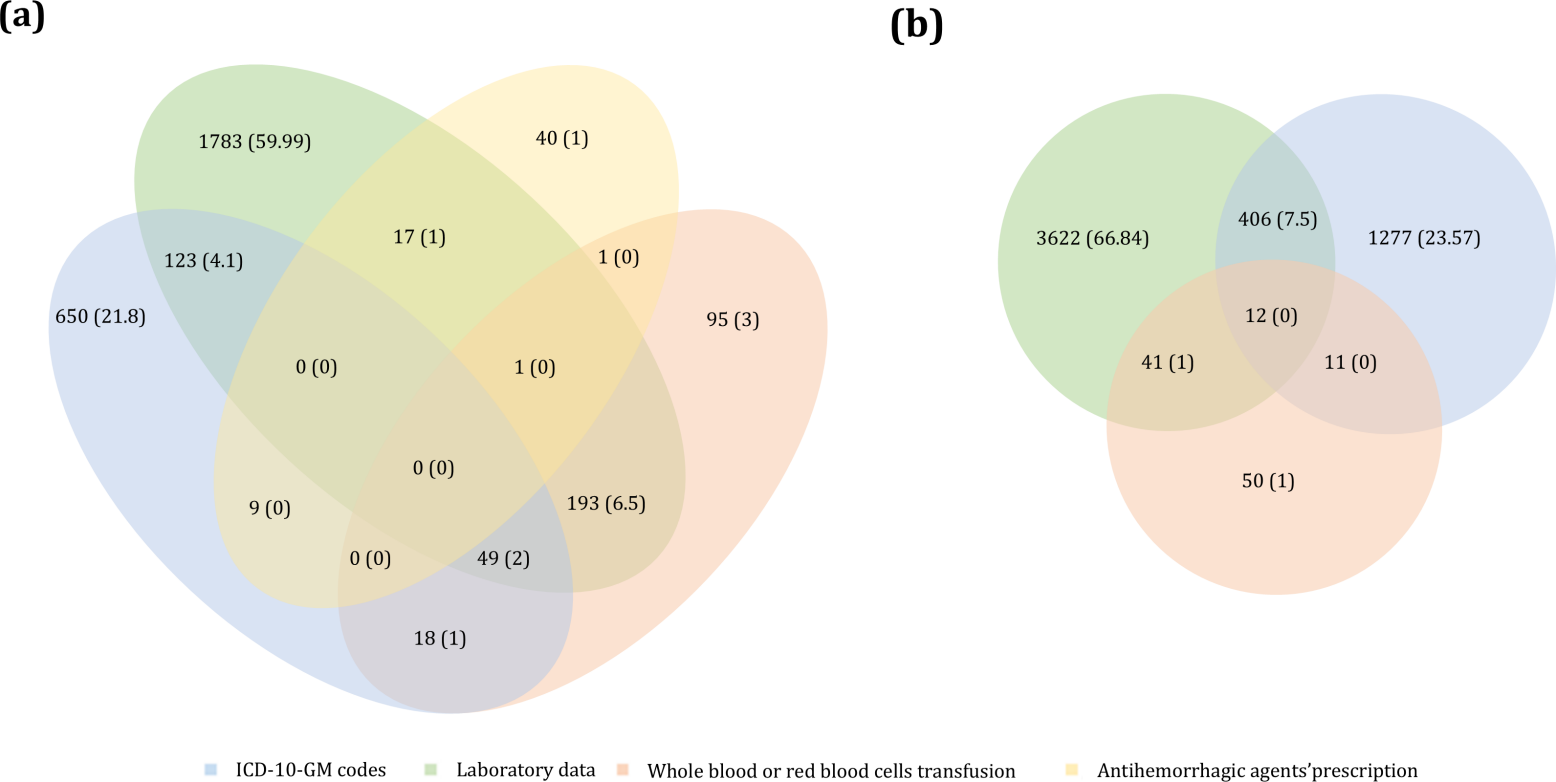
Absolute contribution of structured data sources (laboratory data, *ICD-10-GM* codes, prescription of antihemorrhagic agents, and transfusions) to the detection of (A) major bleeding and (B) clinically relevant nonmajor bleeding. *ICD-10-GM: International Statistical Classification of Diseases, 10th Revision, German Modification*.

Overlap between data sources was limited. Only 12.1% (n=361) of MB stays and 8.7% (n=458) of CRNMB stays were identified by 2 data sources, while detection by all 4 sources occurred in 0% of MB cases and only 0% (n=12) of CRNMB cases (Figure 5). This limited overlap highlights the complementarity, but also fragmentation, of structured data signals.

### Combined Detection Using SDA and NLP (CHUV Only)

Among 7513 CHUV stays with discharge summaries, combining SDA and NLP increased case detection: In total, 39.69% (n=2982) of hemorrhagic cases were detected: 12.2% (n=920) were identified as MB and 27.45% (n=2062) as CRNMB.

For MB cases, 56.6% (n=521) were detected by SDA alone, 19.8% (n=182) by NLP alone, and 23.6% (n=217) by both. For CRNMB cases, 35.1% (n=724) were detected by SDA alone, 48.2% (n=994) by NLP alone, and 16.7% (n=344) by both.

Classification discrepancies were observed between SDA and NLP: 217 cases identified as MB by SDA were reclassified as CRNMB by NLP, and conversely, 81 CRNMB cases by SDA were reclassified as MB by NLP. NLP also enabled the detection of a history of bleeding in 8.5% (n=642) of cases, improving the temporal resolution of hemorrhage onset.

### Internal Validation Using CHUV 2015-2016 Data

The manual review of 754 EMRs identified 276 bleeding cases: 144 MB and 132 CRNMB. Structured laboratory data showed the highest sensitivity (0.58, 95% CI 0.52‐0.64), while *ICD-10-GM* codes had the highest PPV (0.89, 95% CI 0.83‐0.98), and *F*_1_-score (0.60). SDA outperformed NLP in sensitivity (0.77 vs 0.61), but NLP had higher PPV (0.70 vs 0.51) and *F*_1_-score (0.65 vs 0.62). The best performance was achieved by the combined *ICD-10-GM*∪NLP algorithm, with a sensitivity of 0.71 (95% CI 0.66‐0.76), PPV of 0.72 (95% CI 0.66‐0.87), and *F*_1_-score of 0.72. Algorithms combining SDA and NLP yielded the highest sensitivity (0.84), confirming the benefit of multimodal approaches. However, intersection-based algorithms (eg, SDA∩NLP) demonstrated higher specificity at the cost of reduced sensitivity.

Performance metrics for MB and CRNMB subgroups followed similar trends, with reduced sensitivity but high specificity for *ICD-10-GM*–based detection. Table 4 presents a comprehensive summary of all performance metrics, including sensitivity, specificity, PPV, negative predictive value, accuracy, and *F*_1_-score.

**Table 4.** Performance metrics of bleeding detection algorithms compared to manual electronic medical records review (gold standard; n=754)^a^.

	Sensitivity^b^ (95% CI)	Specificity^c^ (95% CI)	PPV^d^ (95% CI)	NPV^e^ (95% CI)	Accuracy^f^ (95% CI)	*F*_1_-score^g^
Bleeding all type (MB^h^ or CRNMB^i^)						
Individual structured data sources						
*ICD-10-GM*^j^	0.46 (0.40‐0.51)	0.97 (0.95‐0.98)	0.89 (0.83‐0.98)	0.75 (0.72‐0.79)	0.78 (0.75‐0.81)	0.60
Laboratory data	0.58 (0.52‐0.64)	0.60 (0.55‐0.64)	0.45 (0.40‐0.64)	0.71 (0.66‐0.75)	0.59 (0.55‐0.62)	0.51
Whole blood or red blood cells transfusion	0.18 (0.14‐0.23)	0.95 (0.93‐0.97)	0.68 (0.57‐0.97)	0.67 (0.63‐0.70)	0.67 (0.63‐0.70)	0.29
Detection algorithms						
SDA^k^	0.77 (0.72‐0.82)	0.58 (0.54‐0.62)	0.51 (0.47‐0.62)	0.81 (0.77‐0.85)	0.65 (0.62‐0.68)	0.62
NLP^l^	0.61 (0.55‐0.67)	0.85 (0.81‐0.88)	0.70 (0.64‐0.88)	0.79 (0.75‐0.82)	0.76 (0.73‐0.79)	0.65
Combined data sources and algorithms						
SDA∪NLP	0.84 (0.79‐0.88)	0.54 (0.49‐0.58)	0.51 (0.47‐0.58)	0.85 (0.81‐0.89)	0.65 (0.61‐0.68)	0.64
SDA∩NLP	0.47 (0.41‐0.53)	0.92 (0.89‐0.94)	0.78 (0.71‐0.94)	0.75 (0.71‐0.78)	0.76 (0.72‐0.79)	0.59
*ICD-10-GM*∪NLP	0.71 (0.66‐0.76)	0.84 (0.80‐0.87)	0.72 (0.66‐0.87)	0.83 (0.80‐0.86)	0.79 (0.76‐0.82)	0.72
*ICD-10-GM*∩NLP	0.31 (0.26‐0.37)	0.99 (0.98‐1.00)	0.95 (0.89‐1.00)	0.71 (0.68‐0.74)	0.74 (0.71‐0.77)	0.47
MB						
Individual structured data sources						
*ICD-10-GM*	0.34 (0.27‐0.42)	0.99 (0.97‐0.99)	0.84 (0.73‐0.99)	0.86 (0.84‐0.89)	0.86 (0.84‐0.88)	0.49
Laboratory data	0.47 (0.39‐0.55)	0.81 (0.77‐0.84)	0.36 (0.30‐0.84)	0.86 (0.83‐0.89)	0.74 (0.71‐0.77)	0.41
Whole blood or red blood cells transfusion	0.22 (0.16‐0.29)	0.97 (0.96‐0.98)	0.66 (0.52‐0.98)	0.84 (0.81‐0.87)	0.83 (0.80‐0.85)	0.32
Algorithms						
SDA	0.72 (0.64‐0.78)	0.79 (0.76‐0.82)	0.45 (0.39‐0.82)	0.92 (0.90‐0.94)	0.78 (0.75‐0.81)	0.55
NLP	0.35 (0.28‐0.44)	0.95 (0.93‐0.96)	0.63 (0.51‐0.96)	0.86 (0.83‐0.89)	0.84 (0.81‐0.86)	0.45
Combined data sources and algorithms						
SDA∪NLP	0.76 (0.68‐0.82)	0.79 (0.75‐0.81)	0.46 (0.39‐0.82)	0.93 (0.91‐0.95)	0.78 (0.75‐0.81)	0.57
SDA∩NLP	0.30 (0.23‐0.39)	0.96 (0.94‐0.97)	0.64 (0.52‐0.97)	0.85 (0.83‐0.88)	0.83 (0.81‐0.86)	0.41
*ICD-10-GM*∪NLP	0.56 (0.48‐0.64)	0.94 (0.92‐0.96)	0.69 (0.60‐0.96)	0.90 (0.87‐0.92)	0.87 (0.84‐0.89)	0.62
*ICD-10-GM*∩NLP	0.14 (0.09‐0.21)	1.0 (0.99‐1.00)	0.91 (0.72‐1.00)	0.83 (0.80‐0.86)	0.83 (0.80‐0.86)	0.25
CRNMB						
Individual structured data sources						
*ICD-10-GM*	0.30 (0.23‐0.39)	0.91 (0.88‐0.93)	0.41 (0.32‐0.93)	0.86 (0.83‐0.88)	0.80 (0.77‐0.83)	0.35
Laboratory data	0.25 (0.18‐0.33)	0.73 (0.70‐0.77)	0.17 (0.12‐0.77)	0.82 (0.79‐0.85)	0.65 (0.61‐0.68)	0.20
Whole blood or red blood cells transfusion	0.03 (0.01‐0.07)	0.96 (0.95‐0.98)	0.15 (0.06‐0.98)	0.82 (0.79‐0.85)	0.80 (0.77‐0.83)	0.05
Detection algorithms						
SDA	0.65 (0.42‐0.58)	0.65 (0.62‐0.69)	0.23 (0.19‐0.69)	0.86 (0.82‐0.89)	0.63 (0.59‐0.66)	0.32
NLP	0.53 (0.45‐0.62)	0.77 (0.74‐0.81)	0.34 (0.28‐0.81)	0.89 (0.86‐0.91)	0.73 (0.70‐0.76)	0.41
Combined data sources and algorithms						
SDA∪NLP	0.66 (0.57‐0.73)	0.56 (0.52‐0.60)	0.24 (0.20‐0.60)	0.88 (0.85‐0.91)	0.58 (0.54‐0.61)	0.35
SDA∩NLP	0.38 (0.30‐0.47)	0.88 (0.84‐0.90)	0.40 (0.32‐0.90)	0.87 (0.84‐0.89)	0.79 (0.76‐0.82)	0.39
*ICD-10-GM*∪NLP	0.60 (0.52‐0.68)	0.75 (0.72‐0.79)	0.35 (0.29‐0.79)	0.90 (0.87‐0.92)	0.73 (0.69‐0.76)	0.44
*ICD-10-GM*∩NLP	0.24 (0.17‐0.32)	0.93 (0.91‐0.95)	0.42 (0.31‐0.95)	0.85 (0.82‐0.87)	0.81 (0.78‐0.83)	0.30

a It should be noted that no patient record contained the variable antihemorrhagic agent for the detection of MB. Consequently, the performance for this variable was not included in the table.

b Sensitivity: proportion of bleeding cases that have been correctly identified.

c Specificity: proportion of nonbleeding-related cases that have been correctly identified.

d PPV: positive predictive value; proportion of bleeding cases among all those classified as bleeding cases by the algorithm.

e NPV: negative predictive value; proportion of nonbleeding-related cases among all those classified as nonbleeding-related cases by the algorithm.

f Accuracy: overall prediction accuracy (ie, the proportion of bleeding and nonbleeding-related cases that the algorithm has correctly identified.

g *F*_1_-score: harmonic mean of the precision and recall (ie, *F*_1_-score = 2 × [recall × precision]/[recall + precision]).

h MB: major bleeding.

i CRNMB: clinically relevant nonmajor bleeding.

j *ICD-10-GM*: *International Statistical Classification of Diseases, 10th Revision, German Modification*.

k SDA: structured data algorithm.

l NLP: natural language processing.

Interrater reliability for manual review of 40 EMRs showed substantial agreement: Fleiss’ Kappa was 0.65 for bleeding detection and 0.61 for MB versus CRNMB classification. Of 276 manually reviewed inpatient stays with bleeding events, 17% (n=48) were attributed to antithrombotic agents. The causal relationship was classified as “certain” in 25% of cases (n=12), “probable/likely” in 23% (n=11), and “possible” in 52% (n=25).

### External Validation Using CHUV 2021-2022 Data

Application of the SDA and SDA+NLP algorithms to the CHUV validation dataset (24054 stays) demonstrated generalizability. The prevalence of MB cases significantly decreased from 10.0% in the 2015‐2016 period to 5.55% (n=1336) in the 2021‐2022 period, while the prevalence of CRNMB cases increased significantly from 14.15% to 16.63% (n=4000). MB in-hospital mortality also rose, from 1.6% to 2.6% (n=616).

Patient characteristics differed significantly between cohorts (Multimedia Appendix 6). Direct oral anticoagulant prescriptions increased from 7.8% to 22.7%, while vitamin K antagonist use decreased from 17.2% to 7.6%. The incidence of elevated INR values >4 declined from 3.3% to 1.8%. Both Charlson and Elixhauser scores increased, reflecting higher comorbidity. Transfusions involving ≤5 units of blood rose from 1.3% to 8.8%. Notably, the proportion of patients receiving ≥3 antithrombotic agents during hospitalization increased fivefold (from 6.7% to 35.0%).

## Discussion

### Principal Findings

To our knowledge, this is one of the first multicenter studies assessing the feasibility and effectiveness of combining structured and unstructured EMR data to detect bleeding cases in older inpatients treated by one or more antithrombotic agents. Across 3 large university hospitals, our SDA identified 8.26% of MB and 15.4% of CRNMB cases. Laboratory variables contributed most to event detection, while *ICD-10-GM* codes alone captured only about one-third of cases, achieving a sensitivity of 0.84 when both data sources were combined. These findings confirm the feasibility of automated bleeding surveillance in real-world hospital data and demonstrate the added value of leveraging free-text information to complement structured data sources.

### Comparison to Prior Work

Our estimated bleeding rates (MB: 8.26% and CRNMB: 15.04%) are consistent with prior hospital-based studies in older adults, which reported MB incidences ranging from 1.8% to 11.3% [35,36] and CRNMB from 3.5% to 13.0% [35,37]. These findings confirm that antithrombotic-related bleeding remains a major cause of ADEs in older populations, associated with increased hospitalization length, morbidity, and mortality [38], highlighting the need for targeted preventive strategies.

The algorithms’ performance varied across structured data sources and aligns with prior research. *ICD-10-GM* codes detected only one-third of MB and CRNMB cases, consistent with previous evidence of underreporting anticoagulant-related bleeding events [39,40]. Yap et al [15] found similarly low sensitivity (16%‐24%) but very high PPV (>0.97), indicating that diagnostic codes are reliable confirmatory markers but poor screening tools. The inclusion of laboratory data markedly improved sensitivity in our SDA model, consistent with findings by Dyas et al [6] and Shung et al [10]. The modest decline in PPV was likely due to false positives generated by hemoglobin thresholds, a limitation noted in earlier work [15].

Detection of CRNMB was more challenging than MB, partly due to broader definitions and lower specificity of *ICD-10-GM* codes and transfusion data, echoing the moderate performance reported by Yap et al [15] (sensitivity 50%‐56%, PPV 43‐50%).

The NLP model contributed substantially to overall detection, with a sensitivity of 61% and PPV of 70%, in line with earlier NLP-based models for bleeding and ADE detection [10,41]. Importantly, only about 20% of events overlapped with those captured by structured data, demonstrating that text analysis retrieves unique clinical insights often missing from coded data. NLP also enhanced temporal resolution by identifying prior bleeding episodes in 8.5% of cases, information generally unavailable from structured data alone. The combined SDA+NLP model achieved high sensitivity (0.84), thereby minimizing the risk of missed events, with only 16% of cases being false negatives. Although this proportion is relatively low, it still represents missed hemorrhagic events that could impact the accuracy of retrospective surveillance and safety signal detection. However, our detection algorithm provides a notable proportion of false positives (49%), which could contribute to alert fatigue in clinical practice and increase the workload associated with unnecessary chart reviews. For real-world deployment, performance thresholds depend on the intended use: for surveillance or signal detection, a sensitivity above 0.80 with PPV above 0.50 is generally acceptable, as false positives can be secondarily reviewed; for clinical decision support, stricter thresholds (eg, PPV≥0.70) are needed to prevent alert fatigue. Improving true positive detection to 70% would strengthen reliability and clinical applicability, potentially through prioritization or triage of clinically significant cases.

External validation revealed a decline in MB prevalence and a concurrent increase in CRNMB and MB-related mortality in the validation dataset (2021‐2022), compared to the CHUV 2015‐2016 dataset. These trends may reflect evolving prescribing patterns, such as increased use of direct oral anticoagulants and reduced use of vitamin K antagonists, and a shift in clinical profiles, with higher comorbidity scores and greater treatment complexity in the more recent cohort. These observations are consistent with the known bleeding risk profiles of antithrombotic agents, direct oral anticoagulants being more frequently associated with gastrointestinal bleeding (CRNMB), and vitamin K antagonists with intracranial bleeding (MB) [42], and underscore the need for dynamic algorithmic models capable of adjusting for changing treatment patterns and patient characteristics [43].

### Strengths and Limitations

This study has several notable strengths. It is one of the first multicenter initiatives to integrate structured and unstructured EMR data for ADE detection in older hospitalized patients. The inclusion of 3 university hospitals provided a large, diverse dataset, while the harmonization of over 1 million clinical variables ensured robust data quality. Algorithms were developed using internationally accepted definitions of MB and CRNMB and validated through manual chart review, ensuring clinical credibility. External validation on a temporally distinct dataset further reinforced reproducibility and robustness.

Several limitations should also be considered.

First, the test dataset (2015‐2016) was relatively dated and spanned only 2 years, reflecting mostly the time-consuming extraction and harmonization process required to merge data from three hospitals before data interoperability infrastructures were implemented. Consequently, it may not entirely capture current clinical practices. Nevertheless, this limitation was mitigated by validating our pipeline on an independent and more recent dataset.

Second, NLP development and validation were performed using CHUV data only and did not take into consideration interinstitutional variations in coding practices, hospital information system architecture and interoperability, clinical documentation standards, or local prescribing patterns, which may limit the generalizability of our findings. To mitigate this, the model was trained on a balanced, manually annotated corpus reviewed by 3 independent physicians. Future studies should externally validate the NLP model on datasets from other French-speaking institutions to confirm its performance and enhance its applicability.

Third, data from 1 hospital (Baden hospital) were excluded due to missing information and harmonization challenges, and CHOP codes could not be extracted from the USZ hospital; this could have led to underestimation of certain bleeding events. Recent efforts have been undertaken to improve data harmonization across sites, which now largely mitigate the harmonization challenges previously encountered.

Fourth, while *ICD-10-GM* code selection was based on international guidelines and expert review, some misclassification may have occurred. This limitation was partly mitigated by manual validation. However, the adoption of a standardized bleeding classification would help overcome this limitation and harmonize bleeding-event categorization across studies.

Fifth, causality assessment between bleeding cases and antithrombotic agents was not formally assessed by our algorithms, as this requires strict criteria and necessitates a comprehensive EMR review. Causality was manually evaluated using the WHO–Uppsala Monitoring Center framework, which provided valuable contextual insights but is resource-intensive. Future work should investigate semiautomated causal-inference tools to scale this process efficiently.

Sixth, structured data were insufficient to capture the timing of bleeding cases prior to admission, as such information is documented in discharge summaries, underscoring the need for unstructured data in ADE detection.

Finally, all participating hospitals were tertiary academic centers with strong data infrastructures and comprehensive documentation practices. While this ensured data reliability and methodological consistency, it may limit the generalizability of our findings to other contexts, such as secondary or community hospitals, or to health systems with different digital maturity levels. Compared to many international settings, Swiss university hospitals operate within a decentralized but highly standardized health care system, characterized by universal coverage and well-developed inpatient services. Future research should evaluate these algorithms in more diverse hospital types and countries to assess their adaptability and scalability beyond tertiary Swiss institutions.

### Future Directions

This study illustrates the value of combining structured and unstructured clinical data to improve the detection of bleeding events in older inpatients exposed to antithrombotic therapy. This integrated approach can enhance pharmacovigilance systems, reduce underreporting, and support timely clinical interventions. Future efforts should expand algorithm coverage to additional unstructured sources (eg, nursing notes and consultation letters), improve clinical documentation practices, and incorporate semiautomated causality assessment tools. Combining these detection models with multivariate risk stratification that integrates patient-specific factors (age, comorbidities, comedications, and clinical service) could enable prioritization of clinically meaningful alerts. Finally, embedding such tools within common data models and privacy-preserving data-sharing infrastructures, such as those promoted by the Swiss Personalized Health Network, could facilitate cross-institutional learning health systems and accelerate artificial intelligence-supported pharmacovigilance in real-world clinical practice.

## Supplementary material

10.2196/77809 Multimedia Appendix 1 International definitions of major bleeding and clinically relevant nonmajor bleeding: a comparative overview.

10.2196/77809 Multimedia Appendix 2 List of synonyms for major bleeding and clinically relevant nonmajor bleeding cases.

10.2196/77809 Multimedia Appendix 3 Interrater reliability assessment among manual reviewers.

10.2196/77809 Multimedia Appendix 4 Causality assessment between antithrombotic treatment and bleeding.

10.2196/77809 Multimedia Appendix 5 Overview of missing values per variable used in major bleeding and clinically relevant nonmajor bleeding algorithms.

10.2196/77809 Multimedia Appendix 6 Comparison of clinical characteristics of Lausanne University Hospital 2015-2016 vs 2021-2022 cohorts.

10.2196/77809 Checklist 1 STROBE checklist.

## Abbreviations

ADE: adverse drug event
CHOP: Swiss Classification of Surgical Procedures
CHUV: Lausanne University Hospital
CRNMB: clinically relevant nonmajor bleeding
EMR: electronic medical record
HUG: Geneva University Hospital
*ICD-10-GM*: *International Statistical Classification of Diseases, 10th Revision, German Modification*
ISTH: International Society on Thrombosis and Haemostasis
KSB: Baden Cantonal Hospital
MB: major bleeding
ML: machine learning
NLP: natural language processing
PPV: positive predictive value
SDA: structured data algorithms
STROBE: Strengthening the Reporting of Observational Studies in Epidemiology
USZ: Zürich University Hospital
