# Supplementary material for: Detection of Antithrombotic-Related Bleeding in Older Inpatients: Multicenter Retrospective Study Using Structured and Unstructured Electronic Health Record Data
Source: J Med Internet Res. 2026 Jan 29;28:e77809. doi: 10.2196/77809 (PMC12854658; doi:10.2196/77809)
Supplement: Multimedia Appendix 1 [file jmir-v28-e77809-s001.docx]

**Appendix 2 - International definitions of Major (MB) and Clinically Relevant Non-Major Bleeding (CRNMB): A comparative overview**

This appendix summarises the selected clinical definitions of MB and CRNMB across major international guidelines, including those from the International Society on Thrombosis and Haemostasis (ISTH)[1, 2], and other pharmacovigilance bodies. The table highlights the heterogeneity in classification criteria and contextualises the rationale behind the operational definitions used in our study. Specific emphasis is placed on clinically actionable variables and data availability within Swiss EMRs.

**Table S2. Overview of international definitions for of major (MB) and clinically relevant non-major bleeding (CRNMB)**

| **Classification** | **Bleeding definition** | **Type of population** |
| --- | --- | --- |
| **Major bleeding definitions** | | |
| **BARC[3]** | **Type 3a:** Overt bleeding and haemoglobin drop of 3-5 g/dL (if the haemoglobin drop is related to the bleeding) or transfusion with overt bleeding.  **Type 3b:** Overt bleeding and haemoglobin drop of 5 g/dL (if the haemoglobin drop is related to the bleeding), cardiac tamponade, bleeding requiring surgical intervention for control, bleeding requiring intravenous vasoactive agents.  **Type 3c:** Intracranial haemorrhage (excluding microbleeds or haemorrhagic transformation, including intraspinal), subcategories confirmed by autopsy or imaging or lumbar puncture, intraocular bleed compromising vision.  **Type 4:** Coronary artery bypass grafting-related bleeding, perioperative intracranial bleeding within 48 hours, reoperation after closure of sternotomy for the purpose of controlling bleeding, transfusion of 5 units of whole blood or packed red blood cells within a 48-hour period, chest tube output 2 litres within a 24-hour period.  **Type 5a:** Probable fatal bleeding, no autopsy or imaging confirmation but clinically suspicious.  **Type 5b:** Definite fatal bleeding, overt bleeding or autopsy or imaging confirmation. | Patients undergoing percutaneous coronary interventions or taking antithrombotic drugs. |
| **GUSTO[4, 5]** | **Severe or life-threatening:** Intracerebral haemorrhage resulting in substantial hemodynamic compromise requiring treatment. | Patients with myocardial infarction undergoing thrombolysis. |
| **ISTH[1, 2]** | Fatal bleeding and/or bleeding in a critical area or organ (e.g. intracranial, intraspinal, intra ocular, retroperitoneal, intra-articular or pericardial bleeds, or intramuscular bleeds with compartment syndrome) and/or bleeding causing a fall in haemoglobin levels by at least 2g/dL (1.24mmol/L) or leading to transfusion of two or more units of whole blood or red cells. | Patients on anticoagulants or anti-platelet drugs. |
| **STEEPLE[6]** | Fatal bleeding, retroperitoneal, intracranial, or intraocular bleeding, bleeding that causes hemodynamic compromise requiring specific treatment, bleeding that requires intervention (surgical or endoscopic) or decompression of a closed space to stop or control the event, clinically overt bleeding, requiring any transfusion of 1 unit or more of packed red blood cells or whole blood, clinically overt bleeding, causing a decrease in haemoglobin of 3 g/dL or more (or, if the haemoglobin level is not available, a decrease in haematocrit of 10% or greater). | Percutaneous Coronary Intervention Patients. |
| **TIMI[7, 8]** | Any intracranial bleeding (excluding microhaemorrhages of less than 10 mm evident only on gradient-echo magnetic resonance imaging); clinically overt signs of haemorrhage associated with a drop in haemoglobin of 5 g/dL or greater; fatal bleeding (bleeding that directly results in death within seven days). | Patients with myocardial infarction or undergoing angioplasty. |
| **ACUITY[9]** | Major bleeding was defined as a haemoglobin drop ≥5 g/dl, intracranial haemorrhage, bleeding requiring surgery or blood transfusion of at least 2 units. | Patients with acute coronary syndrome, including those with or without ST-segment elevation, treated by percutaneous coronary intervention. |
| **CURE[10]** | Life-threatening (fatal, intracranial, requiring surgical intervention, results in substantial hypotension requiring the use of intravenous inotropic agents); haemoglobin decrease ≥ 5g/dL or required ≥4 units of blood; transfusion of 2-3 units of blood, intraocular haemorrhage. | Patients with acute coronary syndrome, including unstable angina and non-ST-segment elevation myocardial infarction. |
| **REPLACE-2[11]** | Intracranial, intraocular, or retroperitoneal bleeding; overt blood loss with haemoglobin decrease > 3g/dL; any haemoglobin decrease > 4g/dL; transfusion of ≥ 2 units of blood products. | Patients undergoing percutaneous coronary intervention. |
| **PLATO[12]** | **Major life-threatening:** Fatal; intracranial; intrapericardial with cardiac tamponade; hypovolemic shock or severe hypotension due to bleeding and requiring pressors or surgery, a decline in the haemoglobin level of 5.0 g per decilitre or more, or the need for transfusion of at least 4 units of red cells.  **Other major:** Significantly disabling (e.g., intraocular with permanent vision loss); associated drop in haemoglobin of 3 to 5g/dL; requiring transfusion of 2 to 3 units of whole blood or red blood cells. | Patients with acute coronary syndrome. |
| **GRACE[13]** | Life-threatening bleeding requiring transfusion of ≥2 units of red blood cells or resulting in an absolute decrease in haematocrit of ≥10% or death, or haemorrhagic/subdural haematoma. | Patients with acute coronary syndrome. |
| **ESSENCE[14, 15]** | Clinically overt bleeding that was fatal; symptomatic intracranial or retroperitoneal haemorrhage; intraocular haemorrhage leading to significant vision loss; decrease in haemoglobin of at least 3.0g/dL; bleeding requiring transfusion of ≥2 units of red blood cells or equivalent of whole blood. | Patients with unstable angina. |
| **Clinically Relevant Non-Major Bleeding definitions** | | |
| **BARC[3]** | **Type 1:** Non-actionable bleeding, not causing the patient to seek unscheduled medical attention or hospitalisation.  **Type 2:** Overt bleeding requiring medical intervention, hospitalisation, or evaluation. | Patients undergoing percutaneous coronary interventions or taking antithrombotic drugs. |
| **GUSTO[4, 5]** | **Moderate:** Requiring a blood transfusion but not resulting in hemodynamic compromise.  **Mild:** Bleeding that does not meet above criteria | Patients with myocardial infarction undergoing thrombolysis. |
| **ISTH[1, 2]** | Any sign or symptom of haemorrhage that does not fit the definition of a major bleed but does meet one of the following criteria: requires medical intervention by a healthcare professional; leads to hospitalisation or increased level of care; or prompts a face-to-face evaluation. | Patients on anticoagulants or anti-platelet drugs. |
| **STEEPLE[6]** | Gross haematuria not associated with trauma (e.g., from instrumentation); epistaxis that is prolonged, is repeated, or requires plugging or intervention; gastrointestinal haemorrhage; haemoptysis; subconjunctival haemorrhage; hematoma > 5cm or leading to prolonged or new hospitalisation; clinically overt bleeding, causing a decrease in haemoglobin of 2 to 3g/d; uncontrolled bleeding requiring protamine sulphate administration. | Percutaneous coronary intervention patients. |
| **TIMI[7, 8]** | Clinically overt (including imaging), resulting in a haemoglobin drop of 3 g/dL to less than 5 g/dL. | Patients with myocardial infarction or undergoing angioplasty. |
| **CURE[10]** | Led to discontinuation of study drug. | Patients with acute coronary syndrome, including unstable angina and non-ST-segment elevation myocardial infarction. |
| **REPLACE-2[11]** | Overt bleeding not meeting criteria for major bleeding | Patients undergoing percutaneous coronary intervention. |
| **PLATO[12]** | Any bleeding requiring medical intervention but not meeting the criteria for major bleeding | Patients with acute coronary syndrome. |
| **ESSENCE[14, 15]** | All other clinically significant bleeding not meeting the definition for major bleeding and that led to interruption of the study drug for at least 24 hours, surgical intervention, or transfusion of ≤ 1 unit of blood. | Patients with unstable angina. |

*BARC: Bleeding Academic Research Consortium; GUSTO: Global Utilization of Streptokinase and t-PA for Occluded Coronary Arteries; ISTH: International Society on Thrombosis and Haemostasis; STEEPLE: Safety and Efficacy of Enoxaparin in Percutaneous Coronary Intervention Patients, an International Randomized Evaluation; TIMI: Thrombolysis in Myocardial Infarction — non-coronary artery bypass grafting–related bleeding; ACUITY: Acute Catheterization and Urgent Intervention Triage Strategy; CURE: Clopidogrel in Unstable angina to prevent Recurrent Events; REPLACE-2: Randomized Evaluation in PCI Linking Angiomax to Reduced Clinical Events-2; PLATO: Platelet Inhibition and Patient Outcomes; GRACE: Global Registry of Acute Coronary Events; ESSENCE: Efficacy and Safety of Subcutaneous Enoxaparin in Non-Q-Wave Coronary Events.*

**REFERENCES**

1. Schulman S, Kearon C. Definition of major bleeding in clinical investigations of antihemostatic medicinal products in non-surgical patients. Journal of thrombosis and haemostasis : JTH. 2005;3(4):692-4.

2. Kaatz S, Ahmad D, Spyropoulos AC, Schulman S. Definition of clinically relevant non-major bleeding in studies of anticoagulants in atrial fibrillation and venous thromboembolic disease in non-surgical patients: communication from the SSC of the ISTH. Journal of thrombosis and haemostasis : JTH. 2015;13(11):2119-26.

3. Mehran R, Rao SV, Bhatt DL, Gibson CM, Caixeta A, Eikelboom J, et al. Standardized bleeding definitions for cardiovascular clinical trials: a consensus report from the Bleeding Academic Research Consortium. Circulation. 2011;123(23):2736-47.

4. An international randomized trial comparing four thrombolytic strategies for acute myocardial infarction. The New England journal of medicine. 1993;329(10):673-82.

5. Rao SV, O’Grady K, Pieper KS, Granger CB, Newby LK, Van de Werf F, et al. Impact of bleeding severity on clinical outcomes among patients with acute coronary syndromes. The American journal of cardiology. 2005;96(9):1200-6.

6. Montalescot G, White HD, Gallo R, Cohen M, Steg PG, Aylward PE, et al. Enoxaparin versus unfractionated heparin in elective percutaneous coronary intervention. The New England journal of medicine. 2006;355(10):1006-17.

7. Chesebro JH, Knatterud G, Roberts R, Borer J, Cohen LS, Dalen J, et al. Thrombolysis in Myocardial Infarction (TIMI) Trial, Phase I: A comparison between intravenous tissue plasminogen activator and intravenous streptokinase. Clinical findings through hospital discharge. Circulation. 1987;76(1):142-54.

8. Bovill EG, Terrin ML, Stump DC, Berke AD, Frederick M, Collen D, et al. Hemorrhagic events during therapy with recombinant tissue-type plasminogen activator, heparin, and aspirin for acute myocardial infarction. Results of the Thrombolysis in Myocardial Infarction (TIMI), Phase II Trial. Annals of internal medicine. 1991;115(4):256-65.

9. Amlani S, Nadarajah T, Afzal R, Pal-Sayal R, Eikelboom JW, Natarajan MK. Mortality and morbidity following a major bleed in a registry population with acute ST elevation myocardial infarction. J Thromb Thrombolysis. 2010;30(4):434-40.

10. Eikelboom JW, Mehta SR, Anand SS, Xie C, Fox KA, Yusuf S. Adverse impact of bleeding on prognosis in patients with acute coronary syndromes. Circulation. 2006;114(8):774-82.

11. Feit F, Voeltz MD, Attubato MJ, Lincoff AM, Chew DP, Bittl JA, et al. Predictors and impact of major hemorrhage on mortality following percutaneous coronary intervention from the REPLACE-2 Trial. Am J Cardiol. 2007;100(9):1364-9.

12. Wallentin L, Becker RC, Budaj A, Cannon CP, Emanuelsson H, Held C, et al. Ticagrelor versus clopidogrel in patients with acute coronary syndromes. The New England journal of medicine. 2009;361(11):1045-57.

13. Moscucci M, Fox KA, Cannon CP, Klein W, López-Sendón J, Montalescot G, et al. Predictors of major bleeding in acute coronary syndromes: the Global Registry of Acute Coronary Events (GRACE). European heart journal. 2003;24(20):1815-23.

14. Cohen M, Blaber R, Demers C, Gurfinkel EP, Langer A, Fromell G, et al. The Essence Trial: Efficacy and Safety of Subcutaneous Enoxaparin in Unstable Angina and Non-Q-Wave MI: A Double-Blind, Randomized, Parallel-Group, Multicenter Study Comparing Enoxaparin and Intravenous Unfractionated Heparin: Methods and Design. J Thromb Thrombolysis. 1997;4(2):271-4.

15. Budaj A, Eikelboom JW, Mehta SR, Afzal R, Chrolavicius S, Bassand JP, et al. Improving clinical outcomes by reducing bleeding in patients with non-ST-elevation acute coronary syndromes. European heart journal. 2009;30(6):655-61.
