# Supplementary material for: Detection of Antithrombotic-Related Bleeding in Older Inpatients: Multicenter Retrospective Study Using Structured and Unstructured Electronic Health Record Data
Source: J Med Internet Res. 2026 Jan 29;28:e77809. doi: 10.2196/77809 (PMC12854658; doi:10.2196/77809)
Supplement: Multimedia Appendix 2 [file jmir-v28-e77809-s002.docx]

**APPENDIX 3 – List of synonyms for MB and CRNMB cases**

**Table S3. List of synonyms for MB and CRNMB cases**

| **Synonyms for MB cases** | |
| --- | --- |
| General terms | Fatal haemorrhage  Intracranial haemorrhage  Intraspinal haemorrhage  Intraocular haemorrhage  Retroperitoneal haemorrhage  Intra-articular haemorrhage  Pericardial haemorrhage |
| Intracranial haemorrhage | Subarachnoid haemorrhage  Intracerebral haemorrhage  Meningeal haemorrhage  Cerebellar haemorrhage  Ventricular haemorrhage  Bridge haemorrhage |
| Intraspinal haemorrhage | Intraspinal haemorrhage  Spinal cord haemorrhage  Spinal subdural haemorrhage  Spinal epidural haemorrhage |
| Intraocular haemorrhage | Hyphema  Choroidal haemorrhage and rupture  Retinal haemorrhage  Vitreous haemorrhage  Choroidal haemorrhage  Optic nerve haemorrhage |
| Retroperitoneal haemorrhage | Morison's space haemorrhage  Broad ligament haemorrhage |
| Intra-articular haemorrhage | Haemarthrosis  Knee haemorrhage  Shoulder haemorrhage |
| Pericardial haemorrhage | Haemopericardium  Cardiac tamponade due to haemorrhage  Haemorrhage in the pericardial sac |
| Intramuscular haemorrhage (with compartment syndrome) | Intramuscular haemorrhage with compartment syndrome  Haemorrhage from the anterior compartment of the leg with compartment syndrome  Forearm deep compartment haemorrhage with compartment syndrome |
| Site unspecified | Hypovolaemic shock  Haemorrhagic shock  Transfusion ≥ 2 red blood cells (or whole blood) with temporal association < 48h of bleeding  Decrease ≥ 20g/L in haemoglobin associated with bleeding |
| **Synonyms for CRNMB cases** | |
| Cutaneous haemorrhage | Ecchymosis  Purpura  Petechiae  Haematoma  Wound bleeding |
| Oral haemorrhage | Gum bleeding  Haemorrhagic stomatitis |
| Gastrointestinal haemorrhage | Rectal haemorrhage  Digestive haemorrhage  Rectal bleeding  Haemorrhoidal bleeding  Gastric bleeding  Duodenal haemorrhage  Bleeding from Barrett's oesophagus  Melena  Black stools  Haematemesis |
| Urogenital haemorrhage | Bladder haemorrhage, non-traumatic  Uterine haemorrhage  Vaginal bleeding  Renal haemorrhage  Intravesical bleeding  Urethrorrhagia |
| ORL haemorrhage | Nose bleed  Epistaxis  Posterior nasal bleeding  Ear haemorrhage  Haemorrhagic pharyngitis |
| Musculoskeletal haemorrhage | Muscle haemorrhage  Haemarthrosis |
| Unspecified site | Unspecified bleeding and bruising  Excessive, abnormal blood loss, blood leakage  Bleeding diathesis  Exacerbations  Spoliative anaemia  Spoliation  Haemoptysis  Haemoptoic  Haematoma  Haematocolpos  Transfusion of 1 red blood cells (or whole blood) with temporal association of 24 to 48 hours of bleeding |
