# Supplementary material for: Detection of Antithrombotic-Related Bleeding in Older Inpatients: Multicenter Retrospective Study Using Structured and Unstructured Electronic Health Record Data
Source: J Med Internet Res. 2026 Jan 29;28:e77809. doi: 10.2196/77809 (PMC12854658; doi:10.2196/77809)
Supplement: Multimedia Appendix 3 [file jmir-v28-e77809-s003.docx]

**APPENDIX 4 – Inter-rater reliability assessment among manual reviewers**

Prior to the full review of the 400 discharge summaries, each of the four independent physician reviewers assessed a preliminary sample of 40 randomly selected summaries to evaluate the robustness and reproducibility of their manual bleeding classification process. Reviewers evaluated the presence of a bleeding event, its classification as MB or CRNMB, and the temporal attribution of the event (e.g., occurring during the hospital stay or prior to admission).

Inter-rater agreement was quantified using Fleiss' kappa (κ) statistic, a standard measure of concordance for categorical ratings among multiple raters. Results indicated substantial agreement:

- κ = 0.65 for the presence or absence of any bleeding event;
- κ = 0.61 for the classification between MB and CRNMB.

These values indicate consistent interpretation of the classification guidelines among reviewers. Discrepant cases were discussed collectively and resolved by consensus, with final annotations used to calibrate the natural language processing (NLP) model. Feedback from this process also informed the refinement of the annotation manual, including clarification of complex cases such as anaemia of multifactorial origin or temporally ambiguous documentation.

This exercise supports the reliability of the gold-standard reference dataset used for training and validating the NLP algorithm described in Appendix 4.

**Table S4. Inter-annotator agreement for bleeding event detection, characterisation, and causality assessment based on Fleiss’ kappa coefficients.**

|  | **Fleiss’ kappa** | **95% CI** | **p-value** | **Level of agreement** |
| --- | --- | --- | --- | --- |
| Bleeding event detection | 0.655 | 0.414-0.896 | <0.05 | Substantial |
| Bleeding type | 0.614 | 0.421-0.806 | <0.05 | Substantial |
| Temporal classification of the bleeding event | 0.571 | 0.395-0.747 | <0.05 | Moderate |
| Death | 0.903 | 0.700-1.000 | <0.05 | Almost perfect |
| Association between the death and the bleeding event | 0.773 | 0.557-0.989 | <0.05 | Substantial |
| Causal relationship between antithrombotic and bleeding | 0.619 | 0.390-0.848 | <0.05 | Substantial |

Bleeding event detection: Measures agreement on the identification of bleeding cases; bleeding type: agreement on categorisation of type of bleeding; temporal classification of the bleeding event: consistency in categorising the timing of the bleeding event as antecedent (bleeding occurring prior to the current hospitalisation), before admission (bleeding identified immediately prior to admission), during hospitalisation (bleeding occurring at any time during hospitalisation), no time frame (no time frame specified); death and association between the death and the bleeding event: assesses consistency in reporting death and its potential association with the bleeding event; causal relationship between antithrombotic drugs and bleeding: agreement on the causal effect of antithrombotic and the bleeding event.
