# Supplementary material for: Detection of Antithrombotic-Related Bleeding in Older Inpatients: Multicenter Retrospective Study Using Structured and Unstructured Electronic Health Record Data
Source: J Med Internet Res. 2026 Jan 29;28:e77809. doi: 10.2196/77809 (PMC12854658; doi:10.2196/77809)
Supplement: Multimedia Appendix 4 [file jmir-v28-e77809-s004.docx]

**Appendix 5 - Causality assessment between antithrombotic treatment and bleeding**

A manual causality assessment was conducted for the identified bleeding cases using a structured tool based on temporal association, biological plausibility, and alternative explanations. Cases were rated as 'certain', 'probable', 'possible', or 'unclassified' in relation to antithrombotic exposure, using per the WHO-Uppsala Monitoring Centre scale[1]. Inter-rater discussion contributed to the standardisation of this classification process.

**Results obtained**: Among the 276 manually reviewed bleeding-related inpatient stays, the evaluators captured information that the bleeding event was attributable to a single antithrombotic drug in 14.5% of cases (n=40). Within this subset, the causal relationship was classified as “certain” in 30.0% of cases (n=12), “probable/likely” in 22.5% (n=9), and “possible” in 47.5% (n=19). In eight stays in which two or more antithrombotic drugs were prescribed, the causal relationship was judged as “probable/likely” in 25.0% of cases (n=2), and “possible” in 75.0% of cases (n=6).

The most frequently implicated antithrombotic drugs were vitamin K antagonists (VKAs), accounting for 50.0% of cases (n=20). Antiplatelet agents and direct Factor Xa inhibitors were each implicated in 30.0% of cases (n=12), followed by unfractionated heparins in 22.5% of cases (n=9), low-molecular-weight heparins in 10.0% of cases (n=4), and other unspecified antithrombotic drugs in 2 cases. No cases involved direct thrombin inhibitors. While 40.8% of inpatient stays involve a single antithrombotic drug, more than half of inpatients stays include two or more drugs, thereby increasing the bleeding risk.

**REFERENCE:**

1. WHO-UMC. The WHO-UMC System for Standardized Case Causality Assessment Upsala, Sweden: World Health Organization - Uppsala Monitoring Centre; 2018 [Available from: <https://who-umc.org/media/164200/who-umc-causality-assessment_new-logo.pdf>.
