# Supplementary material for: Detection of Antithrombotic-Related Bleeding in Older Inpatients: Multicenter Retrospective Study Using Structured and Unstructured Electronic Health Record Data
Source: J Med Internet Res. 2026 Jan 29;28:e77809. doi: 10.2196/77809 (PMC12854658; doi:10.2196/77809)
Supplement: Multimedia Appendix 5 [file jmir-v28-e77809-s005.docx]

**APPENDIX 6 – Overview of missing values per variable used in MB and CRNMB algorithms**

**Table S5: Missing values per variable used in MB and CRNMB algorithms**

| **Variable of interest** | **Missing values**  **n (%)** |
| --- | --- |
| ICD-10 codes MB | 895 (2.48) |
| ICD-10 codes CRNMB | 895 (2.48) |
| Hospital death | 895 (2.48) |
| Laboratory values (Hb) | 2 929 (8.13) |
| CHOP codes transfusion ≤ 5 UI plasma or red blood cells | 34 562 (95.90) |
| CHOP codes transfusion > 5 UI plasma or red blood cells | 895 (2.48) |
| Antidote | 895 (2.48) |
