# Supplementary material for: Detection of Antithrombotic-Related Bleeding in Older Inpatients: Multicenter Retrospective Study Using Structured and Unstructured Electronic Health Record Data
Source: J Med Internet Res. 2026 Jan 29;28:e77809. doi: 10.2196/77809 (PMC12854658; doi:10.2196/77809)
Supplement: Multimedia Appendix 6 [file jmir-v28-e77809-s006.docx]

**APPENDIX 7 – Comparison of clinical characteristics of CHUV 2015–2016 vs 2021–2022 cohorts**

This appendix presents detailed tables of patient demographics, comorbidities, and antithrombotic treatment patterns for both CHUV cohorts. Significant differences include a marked rise in DOAC use, increased transfusion rates, and greater polypharmacy in the later period. A summary paragraph highlights key clinical implications of these trends.

The hospitalised population in the 2021-2022 cohort appeared to be more complex, as reflected by a higher burden of comorbidities and an increased use of antithrombotic therapies. Prescriptions of direct oral anticoagulant (DOAC) increased from 7.8% to 22.7%, while VKA use decreased from 17.2% to 7.6%. The incidence of elevated INR values >4 decreased from 3.3% to 1.8%. Both Charlson and Elixhauser scores increased, reflecting higher comorbidity. Transfusions involving ≤5 units of blood rose from 1.3% to 8.8%. The proportion of patients receiving ≥3 antithrombotic agents during hospitalisation increased fivefold (from 6.7% to 35.0%), indicating a significant evolution toward more intensive antithrombotic therapeutic strategies in the geriatric inpatient population.

**Table S6. Summary of baseline patient characteristics and treatments at CHUV (2015-2016 vs. 2021-2022)**

|  | **CHUV**  2015-2016 (n=7677) | **CHUV**  2021-2022  (n=24054) | **p-value**^1^ |
| --- | --- | --- | --- |
| **Admission Age** | | | |
| Median (min-Max) | 79 (65-99) | 78 (65-107) | **1.E-03** |
| **Sex, n (%)** | | | |
| Male | 3,987 (51.9) | NA |  |
| Female | 3,690 (48.1) | NA |  |
| **Length of stay *(days)*** | | | |
| Median (min-Max) | 9 (1-293) | 7 (1-402) | **1.E-03** |
| **Transfer to intensive care, n (%)** | 467 (6.1) | 1259 (5.2) | **5.E-02** |
| **Inhospital mortality, n (%)** | 345 (4.5) | 7419 (30.8) | **1.E-03** |
| **Comorbidity, n (%)** | | | |
| Chronic renal dysfunction | 2163 (28.2) | 5122 (21.3) | **1.E-03** |
| Dialysis | 176 (2.3) | 516 (2.1) | 0.441 |
| Acute renal dysfunction | 266 (3.5) | 3816 (15.9) | **1.E-03** |
| Chronic liver dysfunction | 294 (3.8) | 649 (2.7) | **1.E-03** |
| Acute liver dysfunction | 145 (1.9) | 328 (1.4) | **1.E-02** |
| Hypertension | 3158 (41.1) | 13140 (54.6) | **1.E-03** |
| Alcohol abuse | 388 (5.1) | 1245 (5.2) | 0.674 |
| Stroke | 813 (10.6) | 1390 (5.8) | **1.E-03** |
| Cancer | 1572 (20.5) | 4447 (18.5) | **1.E-03** |
| Platelet coagulation defect | 496 (6.5) | 1,561 (6.5) | **1.E-03** |
| Anaemia | 1998 (26.0) | 5419 (22.5) | **1.E-03** |
| Risk fall | 2932 (38.2) | 9291 (38.6) | 0.497 |
| Diabetes | 1314 (17.1) | 5045 (21.0) | **1.E-03** |
| Recent myocardial infection | 609 (7.9) | 965 (4.0) | **1.E-03** |
| Low weight | 967 (12.6) | 3728 (15.5) | **1.E-03** |
| Thrombolysis | 180 (2.3) | 271 (1.1) | **1.E-03** |
| Vascular malformation | 153 (2.0) | 800 (3.3) | **1.E-03** |
| **Charlson Comorbidity Index** | | | |
| Median (min-Max) | 0.0 (0.0-9.0) | 2 (1.0-8.0) | **1.E-03** |
| **Elixhauser Comorbidity Index** | | | |
| Median (min-Max) | 0.0 (0.0-6.0) | 1 (0.0-7.0) | **1.E-03** |
| **Antithrombotic categories, n (%)** | | | |
| Direct factor Xa inhibitors | 599 (7.8) | 5705 (23.7) | **1.E-03** |
| Vitamin K antagonists | 1324 (17.2) | 1834 (7.6) | **1.E-03** |
| Heparin group | 5045 (65.7) | 16165 (67.2) | **5.E-02** |
| Direct thrombin inhibitors | 87 (1.1) | 90 (0.4) | **1.E-03** |
| Platelet aggregation inhibitors | 4354 (56.7) | 10918 (45.4) | **1.E-03** |
| Thrombolytics | 15 (0.2) | 68 (0.3) | **1.E-02** |
| Other antithrombotic drugs: Fondaparinux | 212 (2.8) | 310 (1.3) | **1.E-03** |
| **Antidotes, n (%)** | 15 (0.2) | 115 (0.5) | **1.E-02** |
| **Transfusion, n (%)** | 264 (3.4) | 2476 (10.3) | **1.E-03** |
| < 5 UI plasma or red blood cells | 100 (1.3) | 2126 (8.8) | **1.E-03** |
| > 5 UI plasma or red blood cells | 164 (2.1) | 350 (1.5) | **1.E-03** |
| **Number of antithrombotic drugs received during hospitalisation, n (%)** | | | |
| **1** | 4257 (55.5) | 9806 (40.8) | **1.E-03** |
| **2** | 2904 (37.8) | 5827 (24.2) | **1.E-03** |
| **3** | 495 (6.4) | 2854 (11.9) | **1.E-03** |
| **≥ 4** | 21 (0.3) | 5567 (23.1) | **1.E-03** |

*NA: Not available (missing or non-transferred data); n: Total number of recorded measurements for the respective parameter; ^1^ comparison between CHUV and CHUV validation sample using a one-way analysis of variance on ranks (Kruskal-Wallis test) for continuous variables and a Pearson’s chi-squared test for categorial variables.*

**Table S7. Baseline laboratory values at CHUV (2015-2016 vs. 2021-2022)**

|  | **CHUV**  2015-2016 (n=7677) | **CHUV**  2021-2022  (n=24054) |
| --- | --- | --- |
| **Haemoglobin (Hb) (g/L)** |  |  |
| n | 81138 | 45003 |
| Median (min-Max) | 101 (10-223) | 103 (32-206) |
| **Hb nadir (g/L)** |  |  |
| n (%)* | 7207 (8.9) | 8830 (19.6) |
| Median (min-Max) | 110 (10-201) | 109 (32-180) |
| **∆Hb (g/L)** |  |  |
| n | 7052 | 8830 |
| Median (min-Max) | 6 (0-152.6) | 109 (32-180) |
| **∆Hb ≥ 40 (g/L)** |  |  |
| n | 321 | 205 |
| Median (min-Max) | 51 (40-152.6) | 48 (40-131) |
| **20 ≤ Δ Hb < 40 (g/L)** |  |  |
| n | 880 | 1,113 |
| Median (min-Max) | 25 (20-39.5) | 25 (20-39) |
| **INR** |  |  |
| n | 29746 | 17794 |
| Median (min-Max) | 1.1 (1-6) | 1.1 (1.0-6.0) |
| [0-2[, n (%) ** | 24559 (82.6) | 15677 (88.1) |
| [2-4[, n (%) ** | 3756 (12.6) | 1798 (10.1) |
| [4-6], n (%) ** | 977 (3.3) | 319 (1.8) |

*NA: Not available (missing or non-transferred data); n: Total number of recorded measurements for the respective parameter; ∆Hb: Drop in haemoglobin levels observed over a 48-hour period, calculated from a higher initial 'starting' value to a subsequent lower 'arrival' value; *: Percentage relative to the total number of haemoglobins; **: Percentage relative to the total number of INR.*
