# Supplementary material for: Detection of Antithrombotic-Related Bleeding in Older Inpatients: Multicenter Retrospective Study Using Structured and Unstructured Electronic Health Record Data
Source: J Med Internet Res. 2026 Jan 29;28:e77809. doi: 10.2196/77809 (PMC12854658; doi:10.2196/77809)
Supplement: Checklist 1 [file jmir-v28-e77809-s007.docx]

**Table S6: STROBE Statement—checklist of items that should be included in reports of observational studies**

|  | Item No. | Recommendation | Page  No. | Relevant text from manuscript |
| --- | --- | --- | --- | --- |
| **Title and abstract** | 1 | (*a*) Indicate the study’s design with a commonly used term in the title or the abstract | 1 and 4 | Lines 1-3 and 110 |
|  |  | (*b*) Provide in the abstract an informative and balanced summary of what was done and what was found | 2 | Lines 52-56 |
| Introduction | | | |  |
| Background/rationale | 2 | Explain the scientific background and rationale for the investigation being reported | 3-4 | Lines 67-74; 75-89 |
| Objectives | 3 | State specific objectives, including any prespecified hypotheses | 4 | Lines 97-107 |
| Methods | | | |  |
| Study design | 4 | Present key elements of study design early in the paper | 4 | Lines 109-110 |
| Setting | 5 | Describe the setting, locations, and relevant dates, including periods of recruitment, exposure, follow-up, and data collection | 4 | Lines 110-111 and 125-132  Follow-up not applicable |
| Participants | 6 | (*a*) *Cohort study*—Give the eligibility criteria, and the sources and methods of selection of participants. Describe methods of follow-up  *Case-control study*—Give the eligibility criteria, and the sources and methods of case ascertainment and control selection. Give the rationale for the choice of cases and controls  *Cross-sectional study*—Give the eligibility criteria, and the sources and methods of selection of participants | 5 | Lines 130-132 |
|  |  | (*b*) *Cohort study*—For matched studies, give matching criteria and number of exposed and unexposed  *Case-control study*—For matched studies, give matching criteria and the number of controls per case | - | Not applicable. |
| Variables | 7 | Clearly define all outcomes, exposures, predictors, potential confounders, and effect modifiers. Give diagnostic criteria, if applicable | 5-7 | Outcomes: lines 258-261; 268-271; 286-295  Exposures: lines 242-252  Predictors: lines 242-252  Potential confounders and effects modifiers: not applicable |
| Data sources/ measurement | 8* | For each variable of interest, give sources of data and details of methods of assessment (measurement). Describe comparability of assessment methods if there is more than one group | *6-8* | Lines 125-226 |
| Bias | 9 | Describe any efforts to address potential sources of bias | 5-6 | Selection bias: lines 125-128  Measurement bias: lines 158-161  Outcome misclassification bias: lines 170-176  Annotator bias: 186-190 and  Internal validity bias: lines 201-204  Generalisability bias: lines: 212-215 |
| Study size | 10 | Explain how the study size was arrived at | 5 | Lines 125-128  The study size was determined by including all inpatient stays meeting the eligibility criteria during the study periods at the participating hospitals (no sample size calculation was performed). |

Continued on next page

| Quantitative variables | 11 | Explain how quantitative variables were handled in the analyses. If applicable, describe which groupings were chosen and why | 8 | Lines 217-226 |
| --- | --- | --- | --- | --- |
| Statistical methods | 12 | (*a*) Describe all statistical methods, including those used to control for confounding |  |  |
|  |  | (*b*) Describe any methods used to examine subgroups and interactions | 7-8 | Lines 206-213 |
|  |  | (*c*) Explain how missing data were addressed | 6 | Lines 147-150 |
|  |  | (*d*) *Cohort study*—If applicable, explain how loss to follow-up was addressed  *Case-control study*—If applicable, explain how matching of cases and controls was addressed  *Cross-sectional study*—If applicable, describe analytical methods taking account of sampling strategy | - | Not applicable |
|  |  | (*e*) Describe any sensitivity analyses | - | No formal sensitivity analyses were performed; however, external validation on an independent dataset was used to test the robustness of the algorithms. |
| Results | | | | |
| Participants | 13* | (a) Report numbers of individuals at each stage of study—eg numbers potentially eligible, examined for eligibility, confirmed eligible, included in the study, completing follow-up, and analysed | 9 | Lines 251-253 |
|  |  | (b) Give reasons for non-participation at each stage | 6 | Lines 147-150 |
|  |  | (c) Consider use of a flow diagram | - | Not applicable |
| Descriptive data | 14* | (a) Give characteristics of study participants (eg demographic, clinical, social) and information on exposures and potential confounders | 9  20 | Table 1 line 262  Appendix 7 – Table 1 |
|  |  | (b) Indicate number of participants with missing data for each variable of interest | - | Appendix 9 Table 1 |
|  |  | (c) *Cohort study*—Summarise follow-up time (eg, average and total amount) | - | Not applicable |
| Outcome data | 15* | *Cohort study*—Report numbers of outcome events or summary measures over time | - | Not applicable |
|  |  | *Case-control study—*Report numbers in each exposure category, or summary measures of exposure | - | Not applicable |
|  |  | *Cross-sectional study—*Report numbers of outcome events or summary measures | 11-14 | Lines 267 - 345 |
| Main results | 16 | (*a*) Give unadjusted estimates and, if applicable, confounder-adjusted estimates and their precision (eg, 95% confidence interval). Make clear which confounders were adjusted for and why they were included | - | Not applicable |
|  |  | (*b*) Report category boundaries when continuous variables were categorized | 10 | Figure 3 and figure 4 |
|  |  | (*c*) If relevant, consider translating estimates of relative risk into absolute risk for a meaningful time period | - | Not applicable |

Continued on next page

| Other analyses | 17 | Report other analyses done—eg analyses of subgroups and interactions, and sensitivity analyses |  | - | Not applicable |
| --- | --- | --- | --- | --- | --- |
| Discussion | | | | |  |
| Key results | 18 | Summarise key results with reference to study objectives |  | 14-15 | Lines 348-355 |
| Limitations | 19 | Discuss limitations of the study, taking into account sources of potential bias or imprecision. Discuss both direction and magnitude of any potential bias |  | 16-17 | Lines 402-439 |
| Interpretation | 20 | Give a cautious overall interpretation of results considering objectives, limitations, multiplicity of analyses, results from similar studies, and other relevant evidence |  | 14-15 | Lines 329-367 |
| Generalisability | 21 | Discuss the generalisability (external validity) of the study results |  | 15-16 | Lines 356-394 |
| Other information | |  | | |  |
| Funding | 22 | Give the source of funding and the role of the funders for the present study and, if applicable, for the original study on which the present article is based |  | 2 | Line 62 |

*Give information separately for cases and controls in case-control studies and, if applicable, for exposed and unexposed groups in cohort and cross-sectional studies.

**Note:** An Explanation and Elaboration article discusses each checklist item and gives methodological background and published examples of transparent reporting. The STROBE checklist is best used in conjunction with this article (freely available on the Web sites of PLoS Medicine at http://www.plosmedicine.org/, Annals of Internal Medicine at http://www.annals.org/, and Epidemiology at http://www.epidem.com/). Information on the STROBE Initiative is available at www.strobe-statement.org.
